# Mapping unsolved lipidomes accelerates lipid discovery in major bacterial pathogens

**DOI:** 10.1101/2025.11.06.685907

**Published:** 2025-11-07

**Authors:** Yashodhan M. Nair, Aruna R. Menon, Zonghao Lin, Michiel R. L. Vossenberg, Vanisha Munsamy-Govender, David C. Young, Ana M. Xet-Mull, Gregory H. Babunovic, Tan-Yun Cheng, Sahadevan Raman, Kyu Y. Rhee, Jeremy M. Rock, Annemieke de Jong, Adriaan J. Minnaard, Jacob A. Mayfield, David M. Tobin, D. Branch Moody

**Affiliations:** 1 Division of Rheumatology, Inflammation and Immunity, Brigham and Women’s Hospital, Harvard Medical School; Boston, MA 02115, USA.; 2 Department of Molecular Genetics and Microbiology, Duke University; Durham, NC 27710, USA.; 3 Stratingh Institute for Chemistry, University of Groningen; Groningen, 9747 AG, The Netherlands.; 4 Laboratory of Host-Pathogen Biology, The Rockefeller University; New York City, NY 10065, USA.; 5 Division of Infectious Diseases, Weill Department of Medicine, Weill Cornell Medical College; New York City, NY 10065, USA.; 6 Department of Dermatology, Columbia University Irving Medical Center; New York City, NY 10032, USA.; 7 Department of Immunology, Duke University School of Medicine; Durham, NC 27710, USA.

## Abstract

Unlike gene-first approaches to understanding bacterial pathogenesis, molecule-forward discovery can uncover unexpected chemical diversity. Here, new lipidomic analytical methods and quality metrics defined the large scope of unknown lipids in the world’s deadliest pathogen, *Mycobacterium tuberculosis* (Mtb). This map allowed rapid discovery of Mtb lysyldiacylglycerol linked to the biosynthetic gene *lysX*, which controls *in vivo* infection outcomes in moth larvae, mice, guinea pigs, and here, zebrafish. A broader search for orthologous lysyltransferase domains identified the *Staphylococcus aureus* virulence gene *mprF*, where the same lipoamino acid was shown to be a previously unknown biosynthetic product. Thus, lipidomic mapping showed that the cell envelope composition of well-studied bacterial pathogens remains substantially unsolved and offers a new way to generate lists of discoverable lipids to accelerate molecular discovery.

Mtb’s success as the world’s deadliest human pathogen results from evasion and manipulation of human immunity (1). The interface between Mtb and the human host is formed by an extraordinarily complex cell envelope (Fig. 1A) (2). During infection, host nutrients and drugs must pass through two distinct phospholipid and neutral lipid membranes containing at least 22 known major lipid classes (3). Beyond barrier function, genus-specific lipids account for the unique biologic behavior of mycobacteria, including sulfoglycolipids (SGL) that induce cough (4), phthiocerol dimycocerosates (PDIM) that control drug entry (5) and immune evasion (6, 7), mycobactins which scavenge host iron (8), ‘cord factor’ immunogens (9), as well as terpene nucleosides that block phagolysosomal acidification (10).

Recognizing both high lipid complexity in the cell envelope and the incomplete functional annotation of the Mtb genome (11) we hypothesized that uncharacterized lipid virulence factors might exist in large numbers and their discovery could improve our understanding of tuberculosis (TB) disease. Indeed, recent studies have shown that abundant Mtb lipid families escaped discovery despite decades of intensive study of this major pathogen (12, 13). Advances in mass spectrometry, combined with new software and databases for pathogenic bacteria (3, 14) and viruses (15) now allow for rapid and comprehensive detection of a lipidome (16). This technology, which detects mass values for both known and unknown compounds, affords a new opportunity to exclude ions corresponding to known lipids and generate a ‘lipidome of unknowns.’ Such a lipid map might quantitatively circumscribe the scope of undiscovered molecules and accelerate their discovery by focusing on a defined list of targets.

## Qualifying the Mtb lipidome

**Fig. 1.**
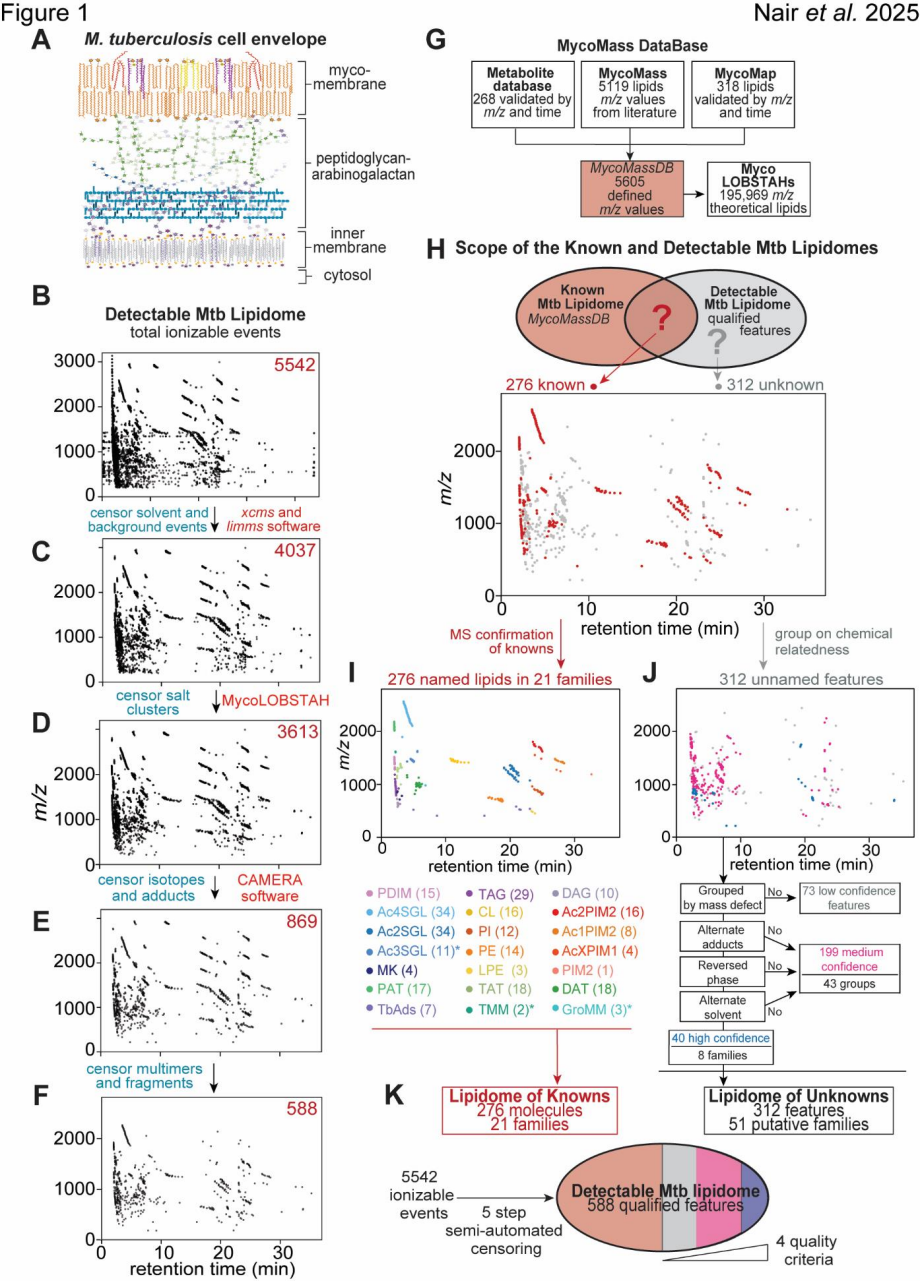
Mapping a lipidome of unknowns in *M. tuberculosis* (**A**) The Mtb cell envelope comprises two lipid membranes. (**B**) The 5542 features of the positive mode lipidome of Mtb H37Rv in biological quadruplicate underwent sequential, semi-automated censoring to remove (**C**) solvent ions, (**D**) salt clusters (**E**) isotopes and alternate adducts, and (**F**) in source multimers and fragments, yielding 588 credentialed features. (**G**) Source databases were updated and combined to create a metabolite and lipid database of defined *m/z* values (MycoMassDB), and a propagated database using LOBSTAHs (21) to generate theoretical lipid variants. (**H**) The 588 qualified features of the ‘detectable lipidome’ were classified as (**I**) 276 features of the ‘lipidome of knowns’, with lead compounds identified by collisional MS and (**J**) 312 features of the ‘lipidome of unknowns’ grouped into lipid families and ranked as high, medium, or low confidence ions based on four criteria: detectable acylforms, the presence of alternate adducts, detection in reversed phase chromatography, and alternate solvent extraction. (**K**) The lipidomes of detectable, credentialed known and unknown lipids were summarized. PDIM, phthiocerol dimycocerosates; Ac4SGL, tetra-acyl sulfoglycolipid; Ac3SGL, triacyl sulfoglycolipid; Ac2SGL, diacyl sulfoglycolipid; MK, menaquinone; PAT, polyacyl trehalose; TAT, triacyl trehalose; DAT, diacyl trehalose; DAG, diacylglycerol; TAG, triacylglycerol; CL, cardiolipin; PI, phosphatidylinositol; PE, phosphatidylethanolamine; LPE, lyso phosphatidylethanolamine; TMM, trehalose monomycolate; GroMM, glycerol monomycolate; Ac2PIM2, diacyl phosphatidylinositol dimannoside; Ac1PIM2 monoacyl phosphatidylinositol dimannoside; AcXPIM1, acyl phosphatidylinositol monomannoside; PIM2, phosphatidylinositol dimannoside. *Lipids with asterisks mass matched to MycoMassDB and were not studied by collisional MS.

We extracted total membrane lipids from matched quadruplicate cultures of Mtb strain H37Rv using chloroform and methanol, excluding glycans, proteins, and nucleic acids. These complex lipid mixtures entered a broadly separating, normal phase high performance liquid chromatography-electrospray ionization time of flight mass spectrometry system (HPLC-ESI-TOF-MS) optimized for high sensitivity and dynamic range (3). We first detected 5542 distinct ion traces known as molecular features, which are linked mass-to-charge ratio (*m/z*), retention time, and intensity values (Fig. 1B).

Detectable but unannotated features in any metabolome may represent valid undiscovered lipids, or MS artifacts from contaminants or redundant detection, where a single valid molecule may be represented as several ions due to modifications that shift the *m/z* (3). These modifications may be biological, such as natural ^13^C-isotopes, or non-biological, such as alternate charged adducts, multimers or fragments formed during electrospray ionization. Chemical investigation of the many observed ions without validation can derail untargeted discovery through considerable wasted effort (17–20). Therefore, we developed a software-based whole organism credentialing pipeline that rapidly appraises data quality to censor artifacts and redundancies, followed by ranking of targets to positively identify features with characteristics of genuine bacterial lipids for further investigation.

Contaminants and idiosyncratic signals were censored by comparison to solvent blanks (Fig. 1C). Salt clusters with low CH content were recognized and censored based on low mass defects using LOBSTAHs (21) (Fig. 1D). Alternate adducts of H^+^, NH_4_^+^, Na^+^ or ^13^C isotopes were censored using CAMERA (22) (Fig. 1E). *In source* lipid aggregates, with high confounding potential given their natural isotopes, and potential acylforms, required manual recognition of multimers of diacyltrehalose (DAT), phosphatidylinositol (PI), phosphatidylethanolamine (PE), phosphatidylinositol mannosides (PIMs) (fig. S1 A to C), recognizable glycerolipid fragments (fig. S1, D to G), and solvent-induced carbamate esters (23) (fig. S1, H to M), yielding 588 unique features.

This conservatively drawn map likely underestimated the true scope of the Mtb ‘pan-lipidome’, as it lacked molecules selectively made by other Mtb strains, or in different growth conditions, trace or unionizable molecules, valid lipids censored by these filters, and high mass lipids above the detection range (~*m/z* 3200). Yet, we intended to determine a focused list as a starting point for molecular discovery with the potential for expansion. Notably, our yield of 588 chemically qualified features from 5542 total features (Fig. 1F) is consistent with previous observations of the high redundancy of detection in MS-based systems biology (17). These ~600 well vetted targets begin to quantitatively circumscribe the Mtb lipidome, and might galvanize discovery approaches to Mtb, depending on what proportion is previously known.

## The unknown lipidome

Further analysis relied on clustering patterns seen in the retention time versus *m/z* plots (Fig. 1H). Considering lipidomics as a distinct sub-discipline of metabolomics, metabolite ions are distributed individually (18, 19), but lipids cluster in groups, where each feature has the same underlying structure and displays the biological signatures of acylforms. Features within a lipid group display characteristic mass differences reflecting their natural biosynthesis in varying alkane (CH_2_, 14.0156 amu) or isoprenoid (C_5_H_8_, 68.0626 amu) series, unsaturation (H_2_, 2.0156 amu), and oxidation (O, 15.9949 amu) patterns. The emergence of clusters and the loss of isolated datapoints during sequential ion censoring (Fig. 1, B to F) served as a general validation of the credentialing pipeline, where artifacts are expected to appear as single ions but true lipids naturally cluster. Further leveraging grouping to visualize and analyze large datasets, we implemented a variant of mass defect filtering (24) whereby the Kendrick mass defect (25), defined as a mass co-efficient transformed by a methylene unit, classifies related acylforms on a horizontal axis (fig. S2, A to B). This made unsaturations, oxygenation, and other modifications apparent on inspection (fig. S2, B to M), and excluded inorganic molecules with low mass defect (fig. S2B).

Next we compared all qualified features to the largest available mycobacterial lipid database, MycoMass (3), which we further augmented with recently reported lipids and 268 validated mycobacterial metabolites to create the MycoMassDB as 5605 known molecules connected to *m/z* values that encompassed both lipidomic and metabolomic space (Fig. 1G, and Dataset S2). After initial analysis connected grouped *m/z* values to named compounds in MycoMassDB, we sought to directly validate matches with collisional MS. To simplify the large scope of effort needed to collide more than 200 matched compounds, we leveraged the phenomenon of lipid family clustering in mass-retention time space. We directly solved 18 lead compounds that defined 18 families, including PDIM, SGLs, acyltrehaloses (AcT), diacylglycerol (DAG), triacylglycerol (TAG), menaquinones (MK), cardiolipins (CL), phospholipids (PI, PE, PIMs) and terpene nucleosides (TbAds) (Fig. 1I and Fig. S3, A to P). Overall, using the methylene transform tool for grouped clusters, we defined the known lipidome of Mtb H37Rv to 276 alkane or unsaturation variants (fig. S2).

Strikingly, 312 qualified features did not match known molecules from MycoMassDB (Fig. 1J), suggesting that unknown lipids might dominate in the Mtb lipidome. Recognizing that even a credentialed lipid list might contain residual artifacts or redundancies (17, 23) we developed rigorous data quality metrics to rank unannotated signals (Fig. 1J). Unknown features were designated as high confidence based on 4 criteria, where ions showed 1.) methylene transformed mass defect grouping patterns indicating acylforms with natural carbon composition and variation; 2.) one molecule [M] as multiple adducts, suggesting a chemical functional group and lack of ionization-dependent fragments; 3.) identification in both reversed and normal phase chromatography, mitigating heteromultimers formed by chromatographic co-elution and shared ionization; and 4.) re-identification with a separate extraction in both chloroform/methanol, and substituted with methylene chloride or deuterated methanol respectively, excluding solvent-induced lipid modification (Fig. 1J, high confidence, *blue*).

154 features in 31 groups met between 1 and 3 and criteria and displayed some features of bacterial lipids, so were considered intermediate confidence and not prioritized for investigation (Fig. 1J, *magenta*). 73 ungrouped low confidence features were not pursued further due to the absence of a lipid alkane series, although they may still be genuine lipids without natural biosynthetic variants (Fig. 1J, *grey*). Overall, conservatively excluding these low and intermediate confidence features, our credentialing pipeline identified a core set of 8 previously unknown families consisting of 40 features that have the highest potential to be undiscovered lipids in this globally important pathogen (Fig. 1K).

## Discovery of lysyldiacylglycerol in Mtb

**Fig. 2.**
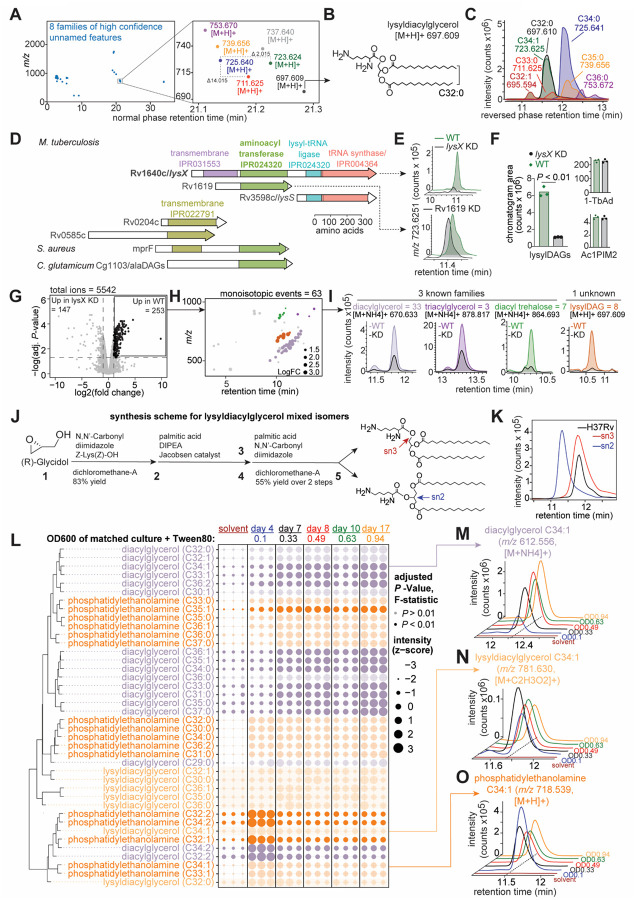
Identification of *lysX*-dependent lysyldiacylglycerol. (**A**) A cluster of high confidence (*blue*) features was shown to have 7 acylforms. (**B**) Collisional MS identified lysyldiacylglycerol (lysylDAG), with (**C**) representative mass chromatograms of acylforms in Mtb H37Rv. (**D**) A search for Mtb genes with homology to lysyltransferases by Interpro (49) subdomain structure identified two candidates, *lysX* and Rv1619. (**E**) Representative single ion chromatograms for the mass of lysylDAG (C34:1, [M+H]+) in total Mtb lipids from CRISPRi knockdowns of *lysX* and Rv1619 show *lysX*-dependence. (**F**) Chromatogram areas for summed lysylDAG acylforms, tuberculosinyl adenosine (*m/z* 540.355 [M+H]+, TbAd), and monoacyl phosphatidylinositol dimannoside (*m/z* 1432.942 [M+NH4]+, Ac1PIM2) analyzed in biological triplicate in the *lysX* KD background. *P*-value determined by pairwise *t*-test. (**G**) Comparative lipidome of *lysX* knockdown against the untreated control in biological triplicate, representative of two independent experiments. A significance threshold using the Benjamini-Hochberg adjusted *P*-value < 0.05 and two-fold change was used to identify *lysX*-dependent features. (**H**) 63 monoisotopic features were classified into three known and one unknown lipid families by grouping acylforms, *m/z* matching, and collisional MS. (**I**) Representative mass chromatograms of *lysX*-dependent families show the relative difference in intensity with *lysX* KD. (**J**) Both lysylDAG with lysine in the sn2 (*blue*) and sn3 (*red*) positions were synthesized in 5 steps. (**K**) Mass chromatograms of the C32:0 (*m/z* 697.609 [M+H]+) acylform of Mtb H37Rv lysylDAG (*black*) co-elutes in the reversed phase with the *sn3* synthetic lysylDAG (*red*) and not the *sn2* synthetic isomer (*blue*). (**L**) The z-score of the intensities of 7 alkylforms of lysylDAG, 17 diacylglycerol (DAG), and 16 phosphatidylethanolamine (PE) in Mtb H37Rv were measured across growth timepoints and plotted as scaled circles with a solvent only negative control. Distributions with a non-significant *P*-value of the F-statistic across all non-solvent pairwise timepoint contrasts, *P* > 0.01, are shown with 25% opacity. Representative of two independent experiments. Representative mass chromatograms show the distribution of (**M**) DAG, (**N**) lysylDAG and (**O**) PE across logarithmic to stationary growth timepoints sampled.

Next we investigated a cluster of seven high quality unnamed features eluting near 21 minutes (Fig. 2A), where monoisotopic *m/z* values differed by 14.015 and 2.015 *m/z*, indicating acylforms of a single underlying core structure. Colliding the C32:0 acylform (*m/z* 697.610) released lysine and diacylglycerol fragments (Fig. 2B and fig. S3Q) (26). Thus, we proposed a structure of lysyldiacylglycerol (lysylDAG) with *sn3* ester-linked lysine appearing as 7 acylforms in Mtb H37Rv (Fig. 2C). These lysine lipoamino acids were distinct from lysine-containing glycerophospholipids (27, 28) based on the absence of phosphate. While these molecules corroborated descriptions in *Mycolicibacterium phlei* (29) and more recently in *Corynebacterium pseudotuberculosis* (30), they were previously unknown in Mtb or the genus *Mycobacterium*.

## Identification of *lysX* as the biosynthetic gene

Amino acyl phosphatidylglycerol synthases (aaPGs) canonically modify phospholipids with cationic amino acids (31), but might have a previously unrecognized function in modifying neutral lipids. For example, *Staphylococcus aureus* MprF catalyzes lysinylation of phosphatidylglycerol (PG) (32), but neutral lipid substrates have recently been identified (33, 34). Mtb contains two paralogous aaPGs: Rv1640c (*lysX*) (35) and Rv1619 (36) (Fig. 2D). CRISPRi gene silencing (37) coupled to HPLC-MS demonstrated that all lysylDAG signals were significantly decreased by the silencing of *lysX* and not Rv1619 (Fig. 2, E and F). By contrast, unrelated lipids 1-tuberculosinyl adenosine (1-TbAd) and monoacyl phosphatidylinositol dimannoside (Ac1PIM2) were unaffected by silencing (Fig. 2F).

Although prior data linked *lysX* to lysyl phosphatidylglycerol (lysylPG), the known MprF product (35), the assignment was not validated by collisional MS or synthetic standards. Further, we could not detect the previously reported phospholipid ion (681.1 *m/z*). Also, another group found that lysylPG persists in *C. pseudotuberculosis* after *lysX* deletion (30). Thus, most data support the assignment of Mtb LysX as an amino acyl diacylglycerol synthase (aaDAGS) (38), rather than a glycerophospholipid modifying enzyme. LysX may be a lysine specific transferase that functionally resembles the corynebacterial enzyme AlaDAGS which catalyzes alanylation of DAG (38).

## Untargeted analysis of *lysX* mutants

Whereas targeted analysis measures a predetermined enzyme product, untargeted lipidomic analysis of *lysX*-silenced bacteria allowed hypothesis-free interrogation of all ionizable Mtb lipids as potential products. Indeed, silencing *lysX* significantly altered 253 of 5542 total features, representing a large lipidomic footprint beyond lysylDAG (Fig. 2G). Censoring isotopes, adducts and multimers, yielded 63 unique events, from which we identified 51 *lysX*-dependent lipids in 4 families (Fig. 2I). Three families were known compounds in MycoMassDB: 33 DAG, 7 DAT, and 3 TAG acylforms. These known lipids showed incomplete depletion (Fig. 2I, *left*), and their structures did not match expected products of an amino acyltransferase, suggesting their signals decreased due to indirect effects of precursor shunting or cell envelope remodeling in response to *lysX* depletion.

In contrast, a single family of eight lipids comprising lysylDAG showed nearly complete loss of MS signal (Fig. 2I, *right*), consistent with being products of the LysX enzyme. To enable structural confirmation by chromatographic co-elution and collisional patterns, we synthesized dipalmitic-lysyl-glycerol initially in two isomeric forms (Fig 2J, and fig. S4A). Mtb lysylDAG matched the *sn3* isomer in chromatographic retention time (Fig. 2K), mass (fig. S4B), and collisional MS spectra (fig. S4, C and D), demonstrating the complete chemical structure of the natural product. Thereafter, we developed a synthesis method for isomerically pure sn3-lysylDAG (fig. S4E).

To test whether lysylDAGs were consistent cell envelope components, we extracted lipids at 5 timepoints across Mtb H37Rv logarithmic to stationary growth phases in media without supplemental oleate or dextrose (fig. S5, A and B). We measured 7 acylforms of lysylDAG, 17 DAG, and 16 PE and analyzed their distributions (Fig. 2L). Next we used all non-solvent pairwise comparisons (fig. S5C) to identify significant (adjusted *P*-value < 0.01 of the F-statistic) lipid variation across growth phases. We observed similar levels of lysylDAG across all timepoints (Fig 2L). This distribution was similar to acylforms of the unrelated glycerolipid PE, and in contrast to the precursor neutral lipid DAG which accumulated in the Mtb cell envelope in liquid culture across growth phases (Fig. 2, M to O). Similarly, in Sauton’s media, where glycerol is the sole carbon source, with and without supplemental lysine, we detected consistent lysylDAG features across 4 growth timepoints, like distributions of PE and DAG (fig. S5, D to I). Thus, lysylDAG biosynthesis was a stable feature of the Mtb cell envelope produced constitutively throughout growth stages, independent of typical precursor flux, media composition, and exogenous lysine.

Lysinylated lipids have a putative mechanistic function in controlling the charge state of the envelope (31). Unlike lysinylated phospholipids that carry an anionic phosphate, lysylDAG has stronger cationic potential that could account for the role of Mtb *lysX* in maintaining Mtb resting membrane potential, resistance to cationic antibiotics vancomycin and polymyxin-B (35), and broader resistance to treatment-relevant antibiotics such as rifampicin and isoniazid (39). Overall, an untargeted ‘lipid-first’ search of the unknown lipidome here identified the downstream metabolite that likely mediates the role of the *lysX* in Mtb virulence and antimicrobial resistance, and expands MycoMassDB by 1 new major class and 30 possible acylforms for future interpretation of altered cell envelope composition by untargeted lipidomics.

## Mapping mycobacterial virulence lipids

**Fig. 3.**
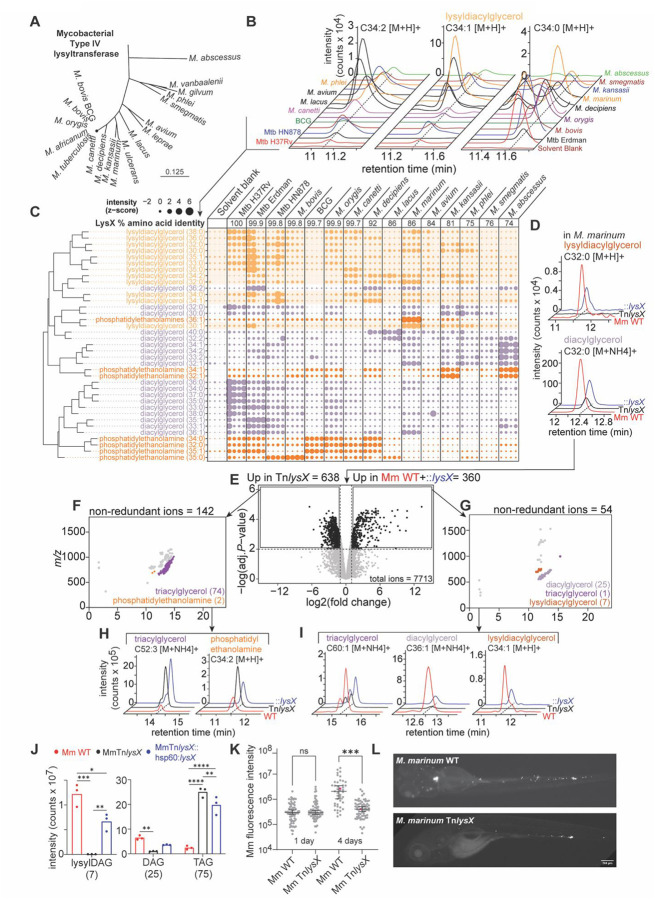
Lysyldiacylglycerol-*lysX*: a conserved mycobacterial virulence pair. (**A**) Subset of a phylogenetic tree of LysX amino acid sequence shows the conservation of *lysX* across mycobacteria, with MprF-domain enzyme classification described previously (38). (**B**) Mass chromatograms show the distribution of representative lysyldiacylglycerol (lysylDAG) acylforms across mycobacteria profiled. (**C**) The z-score intensities of acylforms of lysylDAG, diacylglycerol (DAG), and phosphatidylethanolamine (PE) were plotted as scaled circles in biological quadruplicate lipid extracts from 3 Mtb strains and 12 mycobacteria grown in parallel. (**D**) Mass chromatograms of lysylDAG and DAG in *M. marinum* (Mm) WT, *lysX* transposon mutant (Tn*lysX*) and *lysX* complement (Tn*lysX*::hsp60::*lysX*) show *lysX* dependence. (**E**) A threshold of two-fold change and *P*-value < 0.01 in a compound lipidomic contrast of Mm WT and *lysX* complement against Tn*lysX* identified lipids significantly changed by *lysX* disruption. Strains were grown in biological triplicate, representative of two independent experiments. (**F**) 142 monoisotopic features were enriched in Mm Tn*lysX*. Grouping and lead compound collisional MS identified 74 acylforms of triacylglycerol (TAG) and 2 acylforms of phosphatidylethanolamine (PE). (**G**) 72 monoisotopic features were enriched in Mm WT and ::*lysX*. Grouping and lead compound collisional-MS identified 25 acylforms of DAG, 1 of TAG, and 7 of lysylDAG. Representative single ion chromatograms of (**H**) PE and TAG show enrichment in both Tn*lysX* (*black*) and ::*lysX* (*blue*), whereas (**I**) DAG, lysylDAG, and TAG show enrichment in Mm WT (*red*) and ::*lysX* (*blue*). lysylDAG was absent in the *lysX* mutant. (**J**) Chromatographic areas in the Mm *lysX* lipidome were summed and significant differences were evaluated by two-way ANOVA with Tukey’s post-test (*: *P* < 0.05; **: *P* < 0.01; ***: *P* < 0.001; ****: *P* < 0.0001). (**K**) *M. marinum* burden in zebrafish infection measured using bacterial mCerulean fluorescence. Data shows one representative experiment of 3 biological replicates with 30–60 independent infections per replicate. Median and 95% confidence interval are displayed. Statistical analyses were performed using one-way Welch’s ANOVA followed by a Dunnett’s T3 multiple comparison test of each group to the WT strain (ns: *P* > 0.05; **: *P* ≤ 0.01; ***: *P* ≤ 0.001; ****: *P* < 0.0001). (**L**) Representative images from zebrafish, depicted in (K) in pink, infected with an initial dose of 150–200 fluorescence units of either WT or Tn*lysX* at 4 days post infection.

To determine the species distribution of lysylDAG we reconstructed the phylogeny of major aminoacyl transferases, demonstrating the presence of *lysX* across both disease-causing and environmental mycobacteria (Fig. 3A), consistent with *lysX* as a core mycobacterial gene. We selected representative species across the Mtb complex of highly genetically similar tuberculosis-causing bacteria with different host tropisms, non-tuberculous opportunistic pathogenic mycobacteria, and non-pathogenic environmental mycobacteria, and extracted lipids with equivalent growth conditions in biological quadruplicate. Targeted HPLC-MS lipid analysis of 3 Mtb strains and 12 mycobacterial species identified major C34 acylforms of lysylDAG in every mycobacterium tested with species-specific saturation patterns (Fig. 3B).

While an individual lipid family may be conserved, acylform preferences (40) may hamper attempts to catalogue lipids across species by a single *m/z*. So, we analyzed the acylform distributions of glycerolipids by assessing their relative intensities, using a custom function *mzrtMeta* to align peaks across lipidomic experiments (Materials and Methods). All mycobacteria produced lysylDAG with species-specific acylform patterns. Consistent with PE and DAG, the Mtb complex preferred fully saturated variants of lysylDAG with predominance of the C34:0, C35:0 and C36:0 acylforms. By contrast, environmental and non-tuberculous mycobacteria preferred unsaturated acylforms such as C34:1 and C34:2, that may be consistent with temperature adaptation of cell envelope components to the endothermic host by the Mtb complex common ancestor or the *cfa*-dependent production of tuberculostearic acid from oleic acid by some mycobacteria (41). Therefore, supporting a significant biological role, both *lysX* and lysyldiacylglycerol are broadly conserved across the family Mycobacteriaceae, which includes Mtb and other clinically important non-tuberculous mycobacteria such as *Mycobacterioides abscessus*. In contrast, we also identified reduced but detectable lysyldiacylglycerol levels in the live attenuated vaccine strain Bacille-Calmette Guerin (BCG), the lowest producer of lysyldiacylglycerol across all mycobacteria tested. Moreover, deletion of *lysX*, which is shown here to disrupt lysyldiacylglycerol, previously attenuated Mtb growth *in vivo* or host survival in mice (35), guinea pigs (42) and *C. pseudotuberculosis* outcomes in wax moth larvae (30) in experiments performed with genetic complementation. Thus, the *lysX*-lysylDAG pathway identified here has a non-redundant role in promoting pathogen survival or virulence across evolutionarily diverse actinobacteria-host pairs.

## A *lysX*-lysyldiacylglycerol pathway in *M. marinum*

Beyond demonstrating the presence of lysylDAG in tuberculous and non-tuberculous mycobacteria, phylogenetic analysis showed relatively high production of lysylDAG in *M. marinum* (Mm) (Fig. 3C), a natural pathogen of ectotherms. Mm infection shares key conserved features of tuberculosis pathogenesis that are directly observable in a transparent zebrafish host (43). After generating a *lysX* mutant (Mm:Tn*lysX*) in the candidate orthologue (MMAR_2247) with 86% sequence identity to Mtb LysX (Fig. 3C), we observed loss of lysylDAG, which was restored with complementation (Mm:Tn*lysX*::hsp60::*lysX*) (Fig. 3D). These data established the existence of a lysylDAG pathway in a tractable fish pathogenesis model, setting the stage for whole organism lipidomics and *in vivo* infection experiments.

Untargeted comparative lipidomics of Mm:Tn*lysX* against the wild type (WT) and complemented strains identified ~13% of the lipidome significantly altered by *lysX* disruption (two-fold change, *P* < 0.01) (Fig. 3E). Among 142 non-redundant features enriched in the mutant (Fig 3E), we identified 74 TAG and 2 PE alkylforms, noting a pattern of enrichment in unsaturated acylforms (Fig 3G and fig. S6, A and B). Among 54 non-redundant features reduced in the deletion mutant, 7 lysylDAG acylforms showed complete loss and restoration with complementation. Polar or second site effects were further excluded as disruption of the potential co-operonic downstream gene Rv1639c/MMAR_2446 had no significant effect on lysylDAG levels (Fig. S6F). We also noted partial loss of 25 acylforms of DAG and 1 of TAG, which were restored with complementation (Figure 3H and fig. S6C to E).

Despite the untargeted nature and broad scale of the two analyses in Mtb and Mm, the lipidomic patterns of change after *lysX* disruption were strikingly similar, leading to general conclusions and new questions. First, the complete loss of all acylforms of lysylDAG and its restoration by genetic complementation were most consistent with *lysX* acting as the sole biosynthetic enzyme to catalyze these pathways in both Mtb and Mm. Second, the emergence of changes in DAG and TAG in similar numbers and fold-change parameters in both pathogens represents a highly penetrant and reproducible effect of *lysX* inactivation on neutral lipids other than lysylDAG. While loss of DAG lysinylation through *lysX* deletion predicts accumulation of DAG as a substrate, we observed the opposite. Further, DAG and TAG changed in opposite directions, which, given their usual relationship, predicts DAG to TAG conversion by an unknown mechanism as a means of energy storage (44). *lysX*’s conserved and unexpectedly broad role in governing mycobacterial cell envelope neutral lipid composition suggests an indirect or secondary function beyond that of lysinylation that may contribute to its role in virulence, here uncovered by an untargeted analysis of changes in all detectable downstream lipids following *lysX* disruption independently in pathogenic mycobacteria.

## *lysX* effects on *in vivo* infection of zebrafish

Taking advantage of the tractable and optically accessible nature of zebrafish, we infected larval zebrafish with either wildtype or *lysX* mutant Mm constitutively expressing cerulean fluorescent protein (Fig. 3J). Bacteria from both strains were phagocytosed normally by macrophages within the first hours of infection and resided intracellularly throughout the course of the infection. By 4 days, we observed significant attenuation for the *lysX* mutants deficient in lysylDAG (Fig. 3, J and K), suggesting a role for *lysX* in promoting Mm infection *in vivo*. Along with studies of guinea pig (42), mouse (35) and moth larvae (30), these data provide clear evidence for both the presence and functional importance of *lysX* and its products during infection of evolutionarily diverse hosts.

## Identifying *S. aureus* lysyldiacylglycerol

**Fig. 4.**
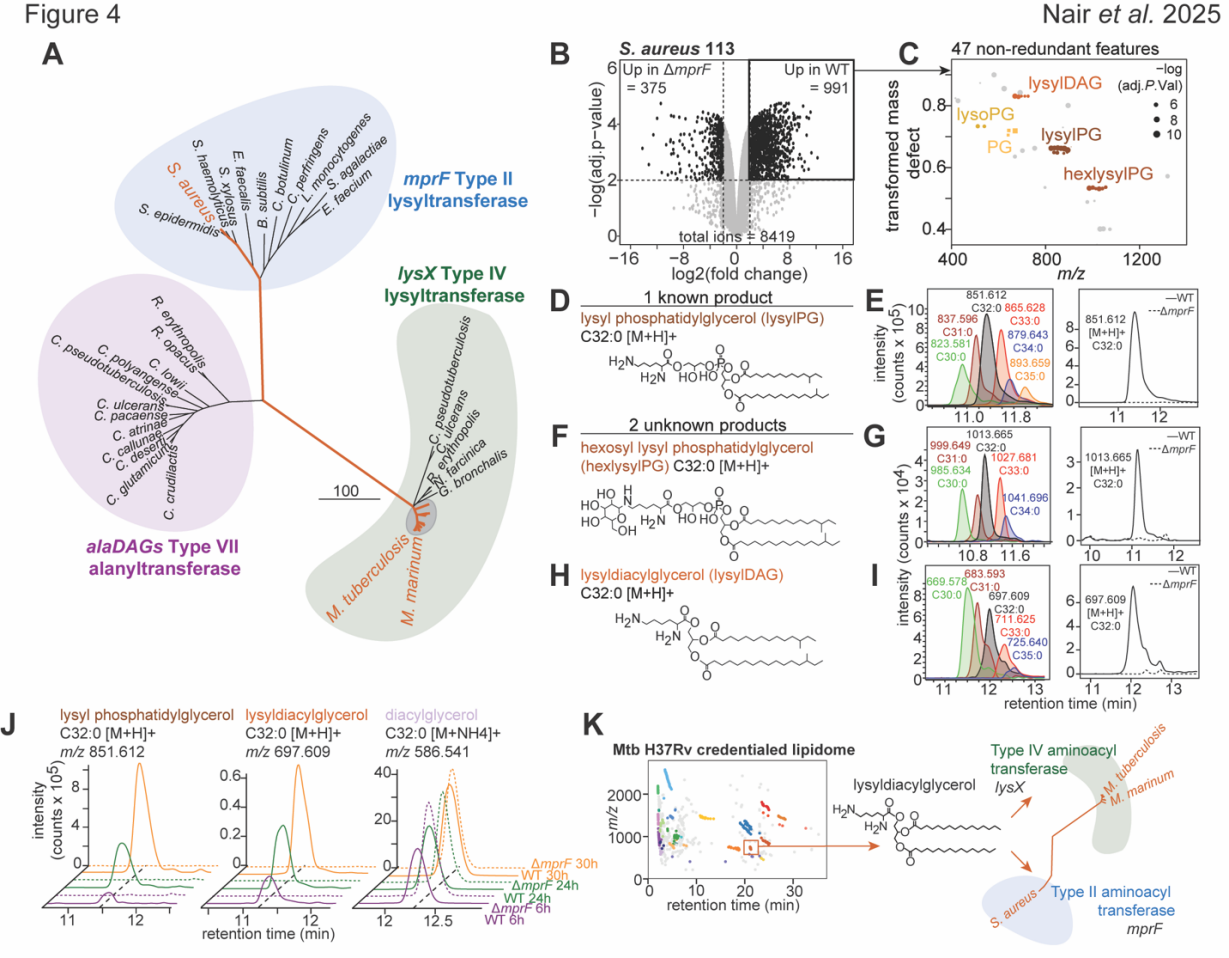
Lysyldiacylglycerol in firmicutes. (**A**) Amino acid sequence phylogeny of major MprF-domain containing proteins in actinobacteria and firmicutes show three clades. (**B**) A comparative lipidome of *S. aureus* 113 WT against the Δ*mprF* mutant was used to identify *mprF*-dependent features in biological triplicate (**C**) Grouping by transformed mass defect, 5 families with > 2-fold change were enriched in the S. aureus WT strain, Benjamini-Hochberg adjusted *P* value < 0.01. (**D**) One family consistent with a known product was identified as lysyl phosphatidylglycerol (lysylPG). (**E**) Representative chromatograms show the lysylPG acylform distribution *S. aureus* WT, *left*, and mass chromatogram showing absence in the *mprF* mutant, *right*. (**F** and **H**) Two previously unknown families were identified by collisional MS as an (*F*) hexosyl-modified lysylPG (hexlysylPG) and (**H**) lysyldiacylglycerol (lysylDAG). (**G** and **I**) Representative mass chromatograms show the acylform distribution of hexlysylPG and lysylDAG in *S. aureus* WT, *left*, and mass chromatogram showing absence in the *mprF* mutant, *right*, consistent with MprF products, and (**J**) show detection of lysylPG, lysylDAG, and DAG across growth phases. (**K**) A summary figure maps the identification of lysylDAG in the ‘lipidome of unknowns’ of Mtb H37Rv, extending this discovery to representative mycobacteria dependent on *lysX* and to *S. aureus* dependent on *mprF*.

LysX is here shown to lysinylate neutral lipids. Such type IV aminoacyl transferases are found throughout Actinobacteria (Fig. 3A) (38). In contrast, two types of related enzymes catalyze other aminoacyl transferase reactions. The corynebacterial Type VII AlaDAGS transfers alanine to neutral lipids. The firmicute Type II enzyme MprF is a lysyltransferase classically producing the abundant firmicute virulence lipid, lysylPG, a phospholipid that mediates broad staphylococcal resistance to cationic antibiotics and antimicrobial peptides (31). To assess possible shared orthology of *lysX*, *mprF*, and *alaDAGS* genes, we constructed a phylogeny by amino acid sequence (38) (Fig. 4A). We mapped *lysX* orthologues beyond mycobacteria as in Figure 3A, defined by a unique transmembrane domain IPR031553 (Fig. 2D), to other actinobacteria including *Rhodococcus*, *Gordonia*, and pathogenic corynebacteria including *Corynebacterium diphtheriae*.

Recognizing the shared lysyltransferase activity of LysX and MprF, and because recent lipid discovery efforts have identified additional MprF products of neutral lipids such as lysyl glucosyl diacylglycerol in *Streptococcus agalactiae* (34) and lysyl diglucosyl diacylglycerol in *Enterococci* (33), we hypothesized that substrate diversity of the MprF enzyme family might be broader than currently known, where undiscovered *mprF*-dependent products might be discoverable by lipidomics tools. We focused on *Staphylococcus aureus*, which is major Gram-positive pathogen that remains a leading cause of death from bloodstream infections and a major source of drug-resistant bacteria in hospital settings (45). Hypothesizing a broader distribution of lysine-modified neutral glycerolipids, we investigated the unsupervised *mprF*-dependent lipidome of *S. aureus*.

Contrasting the *S. aureus* SA113 lipidome against the Δ*mprF* mutant (Fig. 4B), we identified 47 features significantly enriched in the WT strain (Fig. 4C). We used the methylene transformed mass defect tool to identify five lipid families, of which 3 displayed complete signal dependence on *mprF*. First, one of these families was identified as the known *mprF*-product lysylPG both in accurate mass, collisional spectra, and a previously reported acylform preference for odd-chain lipids in staphylococci (46) (Fig. 4, D and E and fig. S7A). Notably, we identified two additional families of lipids with complete *mprF*-dependence and transformed mass defects characteristic of differing oxygenation states (Fig. 4C). Collisional MS identified one of these new families as lysylPG with a hexosyl modification, where neutral loss of 162.050 *m/z* was present in both positive and negative modes (Fig 4F, and fig. S7B). The acylform distribution of this glycerophospholipid in *S. aureus* mirrored that of lysylPG consistent with the derivation therefrom (Fig. 4G). Fulfilling the original hypothesis, we identified a third *mprF*-dependent family as staphylococcal lysylDAG with diagnostic fragments for lysine and neutral losses consistent with odd chain fatty acids (Fig. 4H and fig. S7C). The acylform diversity was distinct from both lysylPG and hexosyl lysylPG characterized here (Fig. 4I), consistent with synthesis from an alternate substrate pool.

To test whether lysylDAG was a consistent feature of the *S. aureus* cell envelope we investigated an *S. aureus* time course dependent lipidome by extracting lipids from both WT and Δ*mprF* mutant at three timepoints and plotting relative lipid intensity by z-score for eight acylforms of lysylPG, 8 PG, 3 lysylDAG and 6 DAG (fig. S7, D to F). Both lysylPG and lysylDAG were consistently detected across all 3 timepoints exclusively in the WT strain in an *mprF*-dependent manner (Fig. 4J). In contrast the neutral precursor, DAG, showed a similar distribution across timepoints and strains (Fig. 4J and fig. S7F). Thus, using both chemical homologies along with gene orthology to drive discovery, this untargeted discovery process extended identification of lysylDAG from Mtb and Actinobacteria to the well-characterized Gram-positive staphylococcal virulence factor *mprF*. Overall, the taxonomic distribution of lysylDAG here identifies a previously unrecognized, broadly distributed component of pathogenic bacterial cell envelopes, dependent on two virulence genes *lysX* and *mprF*, implicated in resistance to antibiotics and human innate immunity.

## Leveraging untargeted lipid discovery

A major limitation of MS-based discovery is that metabolites generate artifactual or redundant signals (17, 18, 47). We quantitatively validate this perspective by showing that in raw Mtb datasets such errors exceed the number of biologically relevant molecules tenfold. Yet, a step-wise qualification process defined specific sources of error and estimated their frequency to pinpoint ~300 unnamed mass-defined features that constitute a ‘lipidome of unknowns.’ A caveat is that we substantially leveraged grouped acylforms as a biosignature of natural molecules that is detectable in high throughput. Thus, while this new approach is applicable to lipids from any source, it may not apply to non-lipid metabolites without repeating mass intervals. Overall, the process of mapping unsolved lipids supports several conclusions.

The search for new molecules is motivated by the expectation that they exist. Arguing against the perspective that decades-long research has already mined the biologically meaningful lipids in Mtb, by extending molecular credentialing efforts (20, 48) we demonstrated that the Mtb lipidome is substantially unsolved. Molecular mapping identified a core set of 40 undiscovered lipids in 8 families that now represent specific high value targets. The first discovery outcome of this process identified lysine-modified neutral lipids produced by both mycobacterial *lysX* and staphylococcal *mprF*, which have broad virulence effects across two bacterial phyla responsible for major global diseases (Figure 4K). Beyond lipoamino acids, by combining genetic manipulation with organism-wide lipidomics we allow unknown downstream chemical mediators of disease to enter studies of bacterial pathogenesis, addressing a mechanistic blind-spot in gene-first analysis. In modern genomics, the complete catalogue of genes is defined, and investigation prioritizes functional phenotyping. By contrast, the scope of unknown metabolites in any system is fundamentally undefined. Here, we argue that sensitive detection of mass-identified unknowns, followed by systematic credentialing and triage, can focus and advance discovery efforts to biologically meaningful outcomes. Finally, by developing an approach to map the ‘dark matter’ of the lipidome, we chart a course to discover further unknown bacterial lipids that mediate diseases which continue to bedevil mankind.

## Supplementary Material

1

Materials and Methods

Figures S1 to S7

References 49 to 58

Data S1 to S13

## Data Availability

All study data, replication markdown, custom functions, and source *xcms* and phenotype objects to replicate all R-based analyses and figures are detailed in the supplementary materials.
