# Supplementary material for: Mapping unsolved lipidomes accelerates lipid discovery in major bacterial pathogens
Source: bioRxiv. 2025 Nov 7:2025.11.06.685907. Preprint. [Version 1] doi: 10.1101/2025.11.06.685907 (PMC12637418; doi:10.1101/2025.11.06.685907)
Supplement: 1 [file NIHPP2025.11.06.685907V1-supplement-1.pdf]

bioRxiv preprint doi: https://doi.org/10.1101/2025.11.06.685907; this version posted November 7, 2025. The copyright holder for this preprint (which was not certified by peer review) is the author/funder, who has granted bioRxiv a license to display the preprint in perpetuity. It is made available under aCC-BY 4.0 International license.

Supplementary Materials for

Mapping unsolved lipidomes accelerates lipid discovery in major bacterial pathogens

Yashodhan M. Nair, Aruna R. Menon, Zonghao Lin, Michiel R. L. Vossenberg, Vanisha Munsamy-Govender, David C. Young, Ana M. Xet-Mull, Gregory H. Babunovic, Tan-Yun Cheng, Sahadevan Raman, Kyu Y. Rhee, Jeremy M. Rock, Annemieke de Jong, Adriaan J. Minnaard, Jacob A. Mayfield, David M. Tobin, D. Branch Moody*

*Corresponding author: bmoody@bwh.harvard.edu

The PDF file includes:

Materials and Methods

Figures S1 to S7

References 49 to 58

Other Supplementary Materials for this manuscript include the following:

Data S1 to S13

bioRxiv preprint doi: https://doi.org/10.1101/2025.11.06.685907; this version posted November 7, 2025. The copyright holder for this preprint (which was not certified by peer review) is the author/funder, who has granted bioRxiv a license to display the preprint in perpetuity. It is made available under aCC-BY 4.0 International license.

Bacterial Strains

Strains used for the feature credentialing pipeline included both *Mycobacterium tuberculosis* H37Rv and the triple auxotrophic *Mycobacterium tuberculosis* H37RvΔ*panCD*Δ*leuCD*Δ*argB* strain mc^{2}7902 was described previously (*50*). Reference bacterial strains used for the intermycobacterial lipid comparative analysis include *Mycobacterium tuberculosis* H37Rv, *Mycobacterium tuberculosis* Erdman, *Mycobacterium tuberculosis* HN878, *Mycobacterium bovis* Ravenel ATCC 35720; *Mycobacterium orygis* 51145; *Mycobacterium canetti* NR-49248/NLA00017120, BEI Resources; *Mycobacterium lacus* sp.Nov NRCM 00-25, BEI Resources; *Mycobacterium decipiens* ATCC TSD-117; *Mycobacterium kansasii* ATCC 12478; *Mycobacterium marinum* ATCC BAA-535, *Mycobacterioides abscessus* sp. *abscessus* Clinical Isolate Taiwan-35; *Mycolicibacterium smegmatis* mc^{2}155; and *Mycolicibacterium phlei* ATCC 11758. *Staphylococcus aureus* SA113 and the Δ*mprF* mutant were previously described (*46*).

Bacterial culture

For lipid extraction related to feature credentialling, Mtb H37Rv was cultured in Difco 7H9 media (BD) supplemented with 10% ADN (albumin 0.5%, dextrose 0.2%, NaCl 0.085% [% w/v], final concentration), 0.2% glycerol [% w/v], at 100 rpm agitation and 37°C in 30 mL PETG Media inkwells (Nalgene). Auxotrophic Mtb H37Rv mc^{2}7902 was grown in 7H9 + 10% ADN + glycerol as above with supplemented PLAM (L-pantothenate 24 mg/L, L-leucine 5 mg/L, L-arginine 200 mg/L and L-methionine 50 mg/L).

For intermycobacterial comparisons, strains were cultured in batches in parallel in either Biosafety Level 3 or 2 laboratories using identical media conditions as above. For *M. marinum*-specific experiments, *M. marinum* was cultured as above but at 120 rpm and 32°C. Cultures were grown in media containing Tween-80 (0.05%, [v/v]) to mid-log phase (OD 0.6-0.8), then subcultured in detergent-free media. Biological replicates of 10 mL cultures were inoculated therefrom in parallel and grown to turbidity in the absence of detergent to a final pelleted cell volume of between 50 - 100 µL/10mL.

To generate CRISPR interference mutants Mtb H37Rv was grown at 37°C in Difco Middlebrook 7H9 broth or on 7H10 agar supplemented with 0.2% glycerol (7H9) or 0.5% glycerol (7H10), 0.05% Tween-80, and 1x oleic acid-albumin-dextrose-catalase (OADC) and 20 µg/mL kanamycin. For lipidomics, Mtb CRISPR interference gene knockdown with anhydrous tetracycline (ATc) 100 ng/mL every 3 days was performed in parallel with uninduced cultures.

For time course lipidomics 7H9 media supplemented with 10% AN was prepared as above without 0.2% dextrose, or Complete Sauton's media (*51*) was prepared as described previously with supplemented 0.02% [w/v] dextrose or 0.02% [w/v] lysine as indicated. For growth phase experiments OD600 was measured in a matched culture containing 0.05% [v/v] Tween-80. Tween free cultures of 50 mL cultures in 125 mL PETG Media inkwells (Nalgene) were extracted in 10 mL at each time point.

S. aureus SA113 and SA113Δ*mprF* were cultured in Tryptic Soy Broth as described previously (*46*). For time course lipidomics *S. aureus* SA113 was cultured in biological triplicate in 5mL Luria Bertani (LB; Bacto Tryptone 10g/L, Gibco BD; Bacto Yeast Extract 5 g/L, Gibco BD; NaCl 10 g/L, Fisher) media in 14 mL vented snap-cap tubes (Corning) at 200 rpm and 37°C.

Lipid extraction

All organic solvents were HPLC-grade or Optima® (Fisher). Mycobacterial cultures of 10 mL were pelleted at 4000 rpm (Thermo Sorvall Legend RT centrifuge; Rotor, Sorvall Heraeus 75006445), washed and resuspended in phosphate buffered saline (PBS; pH 7.4, Gibco) twice and resuspended in 2:1 [v/v] methanol:chloroform in 15 mL conical borosilicate glass tubes (Kimble) with phenolic caps (Kimble) for

bioRxiv preprint doi: https://doi.org/10.1101/2025.11.06.685907; this version posted November 7, 2025. The copyright holder for this preprint (which was not certified by peer review) is the author/funder, who has granted bioRxiv a license to display the preprint in perpetuity. It is made available under aCC-BY 4.0 International license.

inactivation. After removal from the BSL-3 or BSL-2 by approved inactivation protocols, bacteria were agitated for 1 h at 40 rpm on an orbital shaker. The cells were pelleted at 3000 rpm and the supernatant transferred to clean round-bottomed 15 mL glass tubes (Pyrex). A second extraction with 1:1 [v/v] methanol:chloroform was performed and the resulting supernatant after agitation and centrifugation as above was pooled. Lipids were dried under N_2-gas (N-EVAP 111, Organomotion) or by GeneVac (Low BP Mixture setting, 30°C; EZ-2, SP Scientific), resuspended in 1:1 [v/v] methanol:chloroform and sonicated for 3 min at 60 sonics/min (Branson 5510 Sonicator). The undissolved particles were pelleted at 3000 rpm. The supernatant was transferred to pre-weighed 4 mL amber vials (Supelco) with teflon-lined caps (Supelco), dried under N_2-gas and weighed (Mettler Toledo, XP205). Lipid yield ranged between 1-2 mg/10 mL culture. Lipid extracts were resuspended in 1:1 [v/v] methanol:chloroform, normalized to 1 mg/mL and stored at -20°C.

For lipid extraction using alternate solvents deuterated methanol (d4-methanol) and methylene chloride we performed two parallel extracts of Mtb H37Rv auxotroph mc^{2}7902 as above, replacing methanol with d4-methanol [v/v], and chloroform with methylene chloride [v/v], respectively.

For lipid extraction from *S. aureus*, cells were washed twice with 20 mM acetate buffer, pH 4.5 as described previously (*46*), thereafter inactivation with 2:1 [v/v] methanol:chloroform, and subsequent lipid extraction proceeded as above for mycobacterial lipids.

Liquid Chromatography and Mass Spectrometry

An optimized method for mass spectrometry of mycobacterial lipids was described previously (*3*). Briefly, an Agilent 6530 Accurate-Mass QTOF coupled to a 1220 Infinity series HPLC system was used for normal phase chromatography with an Inertsil diol column (3 µm, 2.1 x 150 mm) and a 3 µm cartridge guard column (3 x 10 mm x 2). Lipids were resuspended at 1 mg/mL in solvent B (hexanes:isopropanol, 70:30 [v/v], 0.02% [m/v] formic acid, 0.01% [m/v] ammonium hydroxide) in 2 mL amber screw vials (Agilent) with 250 µL pulled point conical glass insert (Agilent) and PTFE/RS blue screw caps (9 mm, Agilent). 20 µg of total lipid per sample were injected and eluted by binary gradient of 0.15 mL/min from 0% to 100% solvent A (isopropanol:methanol, 70:30 [v/v], 0.02% [m/v] formic acid, 0.01% [m/v] ammonium hydroxide): 0–10 min, 0% A; 17–22 min, 50% A; 30–35 min, 100% A; 40–44 min, 0% A, followed by additional 5 min 0% A postrun.

For reversed phase chromatography we used an InfinityLab Poroshell 120 EC-C18 (3.0 x 50 mm, 1.9 µm, Agilent) with a 3 mm guard column (2.7 µm, Agilent). Lipids were resuspended at 1 mg/mL in solvent A (9:1 methanol:water [v/v], and 2 or 10 mM NH_4HCOO), and eluted by binary gradient of 0.15 mL/mL from 0% to 100% solvent B (9:1 1-propanol:cyclohexane [v/v], 3 mM NH_4HCOO): 0-10 min, 0% B; 10-15 min, 100% B; 15-23 min, 100% A. After chromatographic separation, samples were ionized by electrospray with a gas temperature of 325°C, 5 L/min drying gas, 15 psi nebulizer pressure, and 5500 V with time of flight detection within a range of 200 to 3200 *m/z*. For the *lysX* and Rv1619 CRISPRi knockdown lipidomics we used an InfinityLab Poroshell HPH-C18 column (2.1 x 50 mm, 2.7 µm, Agilent) and a 4.6 x 5 mm guard column (2.7 µm, Agilent) with 0.1% [v/v] ammonium hydroxide added to both reversed phase mobile phases.

Collision-induced MS was performed with an optimized voltage for each lipid family ranging from 15-175 V in positive and/or negative modes as indicated in , Fig. S3. A narrow window for parent ion isolation (1.3 *m/z*) was used with parent ion identification by chromatographic retention time and *m/z* within 10 ppm of the initial detected *m/z*.

Processing of MS data

bioRxiv preprint doi: https://doi.org/10.1101/2025.11.06.685907; this version posted November 7, 2025. The copyright holder for this preprint (which was not certified by peer review) is the author/funder, who has granted bioRxiv a license to display the preprint in perpetuity. It is made available under aCC-BY 4.0 International license.

Chromatograms and mass spectra were generated from raw data (.d) using Mass Hunter Qualitative Analysis software (Agilent) and images were formatted for publication in Adobe Illustrator. Centroided data as .mzXML files were obtained from raw data using *msconvert* (*52*) Peak picking was performed using *xcms* (*53*). Standard parameters included a minimum filter of 200 *m/z,* signal-to-noise threshold of 10, peakwidth of 20-120, and *m/z* filter of 200 to 3200 *m/z*. Arguments such as bandwidth (5-30) and ppm (5-10) were optimized to the experiment and chromatography phase. See Data S1 R-Markdown script for a detailed breakdown of *xcms* parameters used for each experiment, including the detailed sequential credentialing pipeline for unknown lipids, the methylene transform analysis and plots, as well as *ggplot2* scripts used to generate each R-based main and supplemental panel. Included is an additional R-markdown of the custom functions used to access *xcms* and CAMERA that were not included in the *limms* package (*16*). Statistical analysis not using *limms* used Graphpad Prism (v10.3).

Briefly, we used *limms* for differential abundance analysis and imputing local intensity minima to enable pairwise or compound statistical contrasts, as described previously (*16*). Thereafter, linear models were tested to generate Benjamini-Hochberg adjusted *P*-values reported for the contrast. For the credentialing pipeline, contaminants and idiosyncratic signals were identified by statistical comparison to solvent-only HPLC-MS control runs. Ions that did not meet a significance threshold of $P < 0.05$, fold change > 2 were censored. The core lipid structures in MycoMassDB were propagated to ~200,000 theoretical variants using LOBSTAHs (Data S2) (*21*). The MycoLOBSTAHs database worked best as an indicator that an observed masses contained C, H, O, N, P, or S atoms in a ratio typical of a lipid rather than an annotation tool, and was used to identify inorganic ions and salt clusters due to their low CH content. Automated mass matching was performed using the dbMatch function within *limms* and MycoMassDB or MycoLOBSTAHs databases to 10 ppm and no retention time matching. Adducts of H^+, NH_4^+, Na^+ or ^{13}C isotopes were identified using the R-package CAMERA using default parameters in the positive mode polarity (*22*). Identifying *in source* lipid aggregation required manual recognition of multimers of diacyl trehalose (DAT), phosphatidylinositol (PI), phosphatidylethanolamine (PE), phosphatidylinositol mannosides (PIMs) and glycerolipid fragments, as seen in , Fig. S1. To generate the methylene transformed co-efficient, or the CH_2 Kendrick mass defect, the detected *m/z* of each lipid was multiplied by 14/14.01565 and subtracted by the floor integer of the transformed mass to give the transformed mass defect (Equation 1) (*25*). Lipids were grouped based on their retention time (± 3 min) and transformed mass defect coefficient (±0.025).

$$Kendrick\ mass\ defect\ (\mathrm{CH_2}) = \left(Detected\ Mass\ (\mathrm{m/z}) \times \frac{Nominal\ mass\ (\mathrm{CH_2})}{Exact\ mass\ (\mathrm{CH_2})}\right) - \left\lfloor Detected\ Mass\ (\mathrm{m/z}) \times \frac{Nominal\ mass\ (\mathrm{CH_2})}{Exact\ mass\ (\mathrm{CH_2})} \right\rfloor$$

$$Kendrick\ mass\ defect\ (\mathrm{CH_2}) = \left(Detected\ Mass\ (\mathrm{m/z}) \times \frac{14}{14.01565}\right) - \left\lfloor Detected\ Mass\ (\mathrm{m/z}) \times \frac{14}{14.01565} \right\rfloor$$

Equation 1. Methylene transformed mass defect or CH_2 Kendrick mass defect

<u>Domain architecture of lysyltransferases in Mtb</u>

Using the Uniprot entries for Mtb *lysX*/Rv1640c (P9WFUZ), *S. aureus mprF* (Q2G2M2) and *C. glutamicum alaDAGS*/cg1103 (P35867) genes with shared Interpro classified domains were identified in Mtb (*49*). The data on gene and domain length were obtained from Interpro. Visualization used the R-package *gggenes* function *geom_gene_arrows*.

<u>Generation of Rv1619 and *lysX* CRISPRi knockdown strains</u>

bioRxiv preprint doi: https://doi.org/10.1101/2025.11.06.685907; this version posted November 7, 2025. The copyright holder for this preprint (which was not certified by peer review) is the author/funder, who has granted bioRxiv a license to display the preprint in perpetuity. It is made available under aCC-BY 4.0 International license.

Individual CRISPRi plasmids were generated following the protocol outlined in (*54*) using Addgene plasmid #163631. Briefly, the CRISPRi plasmid backbone was digested with BsmBI-v2 (NEB #R0739L) and subsequently gel purified. sgRNAs were designed to target the non-template strand of the target gene ORF. For each sgRNA, two complementary oligonucleotides with compatible sticky-end overhangs were annealed and ligated into the BsmBI-digested plasmid backbone using T4 DNA ligase (NEB #M0202M). For lysX/Rv1640c the top oligonucleotide was 5'-GGGAGCCCAGAACTCCCGATAGCCCAGCA-3' and bottom oligonucleotide was 5'-AAACTGCTGGGCTATCGGGAGTTCTGGGC-3', predicted strength 0.91 (PAM 5'-CCAGAAC-3'). For Rv1619 the top oligonucelotide was 5'-GGGAGCCGCGAAGTCGGCGACCAGCTG-3' and the bottom oligonucleotide was 5'-AAACCAGCTGGTCGCCGACTTCGCGGC-3', predicted strength 0.96 (PAM 5'-GGGGAAT-3'). Ligation products were transformed into NEB 5-alpha chemically competent cells and selected on LB + kanamycin (50 µg/mL). Individual colonies were picked and the correct sgRNA sequence was confirmed by Sanger sequencing before subsequent transformation into *M. tuberculosis* H37Rv.

For transformations of *M. tuberculosis* H37Rv, cultures of at least 20 mL were grown to late logarithmic phase (OD600 of 0.8–1.0) and cells were pelleted at 4,000 × g (room temperature) for 10 min. The cell pellet was washed three times in sterile 10% glycerol. The washed cells were then resuspended in 10% glycerol in a final volume of 5% of the original culture volume. For each transformation, 100 ng plasmid DNA and 100 µL of electrocompetent mycobacteria were mixed and transferred to a 2 mm electroporation cuvette (Bio-Rad #1652082). Electroporation was performed using the Gene Pulser X cell electroporation system (Bio-Rad #1652660) set at 2500 V, 700 Ω, and 25 µF. Bacteria were recovered in 7H9 for 24 hr. After the recovery incubation, cells were plated on 7H10 with 20 µg/mL kanamycin. Plates were subsequently incubated at 37°C for 14 -21 days. Individual colonies were picked and the correct sgRNA sequence was confirmed by Sanger sequencing.

<u>Synthesis of sn3-lysyldiacylglycerol</u>

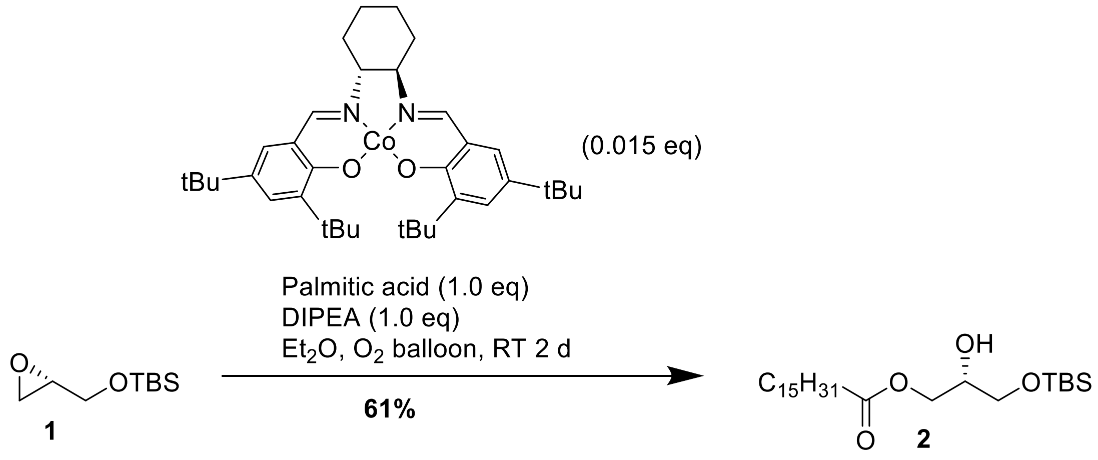

(*S*)-3-((tert-Butyldimethylsilyl)oxy)-2-hydroxypropyl palmitate **2**

An oven-dried flask was charged with palmitic acid (6.9 g, 26.9 mmol, 1.0 eq), (*R,R*)-Co[salen] (240 mg, 0.398 mmol, 1.5 mol%) and Et_2O (30 mL). The flask was equipped with an oxygen balloon that provided a flow of O_2 directly into the resulting solution. The resulting mixture was stirred for 30 min, during which the reaction turned dark brown. Afterwards, DIPEA (4.6 mL, 26.5 mmol, 1.0 eq) was added, and after 5 min of stirring, **1** (5.6 mL, 26.5 mmol). The resulting reaction was stirred at RT, until after 2 d, TLC (95 : 5 pentane : Et_2O) indicated near-complete consumption of **1**. The reaction was subsequently concentrated, dispersed onto celite and purified using flash

bioRxiv preprint doi: https://doi.org/10.1101/2025.11.06.685907; this version posted November 7, 2025. The copyright holder for this preprint (which was not certified by peer review) is the author/funder, who has granted bioRxiv a license to display the preprint in perpetuity. It is made available under aCC-BY 4.0 International license.

column chromatography on silica (pentane : Et_2O 95 : 5 to 90 : 10) to obtain **2** as a white wax (7.2 g, 16.3 mmol, 61%).

^{1}H NMR (400 MHz, Chloroform-*d*) δ 3.85 (dd, *J* = 11.9, 3.2 Hz, 1H), 3.66 (dd, *J* = 12.0, 4.8 Hz, 1H), 3.09 (tt, *J* = 4.0, 2.9 Hz, 1H), 2.77 (dd, *J* = 5.1, 4.0 Hz, 1H), 2.64 (dd, *J* = 5.2, 2.7 Hz, 1H), 2.34 (t, *J* = 7.5 Hz, 2H), 1.63 (p, *J* = 7.4 Hz, 2H), 1.25 (d, *J* = 2.1 Hz, 24H), 0.90 (s, 9H), 0.89 – 0.83 (m, 2H), 0.08 (d, *J* = 3.6 Hz, 6H).

^{13}C NMR (101 MHz, cdcl$_3$) δ 179.22, 63.71, 52.44, 44.49, 33.90, 31.92, 29.69, 29.68, 29.66, 29.65, 29.64, 29.59, 29.43, 29.36, 29.24, 29.06, 25.86, 24.68, 22.69, 14.12, -5.32, -5.36.

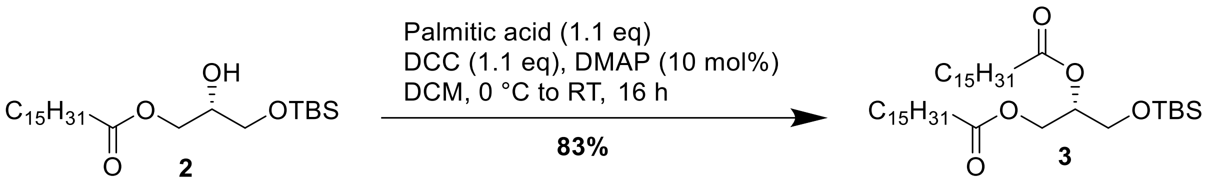

(*S*)-3-((tert-Butyldimethylsilyl)oxy)-2-(palmitoyloxy)propyl palmitate **3**

An oven-dried flask under N_2-atmosphere was charged with **2** (8.06 g, 18.1 mmol), palmitic acid (5.21 g, 20.3 mmol, 1.1 eq), anhydrous DCM (120 mL) and DMAP (221 mg, 1.81 mmol, 10 mol%). The resulting solution was cooled to 0 °C over 5 min before addition of DCC (4.20 g, 20.3 mmol, 1.1 eq). The reaction was subsequently brought to RT and stirred for 16 h before TLC (98 : 2 pentane : Et_2O) indicated near-complete conversion of the starting material. The reaction was subsequently concentrated *in vacuo*, dispersed onto celite and purified using flash column chromatography on silica (pentane : Et_2O 99 : 1 to 90 : 10) to provide **3** as a white wax (10.2 g, 15.0 mmol, 83%).

^{1}H NMR (400 MHz, cdcl$_3$) δ 5.05 (dqt, *J* = 9.2, 5.7, 2.8 Hz, 1H), 4.32 (ddt, *J* = 11.9, 4.4, 2.2 Hz, 1H), 4.13 (ddt, *J* = 13.1, 6.8, 3.4 Hz, 1H), 3.69 (ddd, *J* = 5.3, 3.7, 1.6 Hz, 2H), 2.27 (tdt, *J* = 7.1, 5.1, 2.4 Hz, 4H), 1.59 (dtq, *J* = 10.6, 7.5, 4.8 Hz, 4H), 1.23 (d, *J* = 4.9 Hz, 48H), 0.92 – 0.80 (m, 15H), 0.05 – 0.00 (m, 6H).
^{13}C NMR (101 MHz, cdcl$_3$) δ 173.36, 71.64, 62.40, 61.43, 34.30, 34.12, 31.90, 29.67, 29.64, 29.60, 29.45, 29.34, 29.26, 29.10, 29.07, 25.71, 25.69, 24.92, 24.89, 22.66, 18.15, 14.06, -5.57.
HRMS (ESI+) m/z calcd for $[M+Na]^+$ $C_{41}H_{82}O_5SiNa$ = 705.5824; found 705.5821

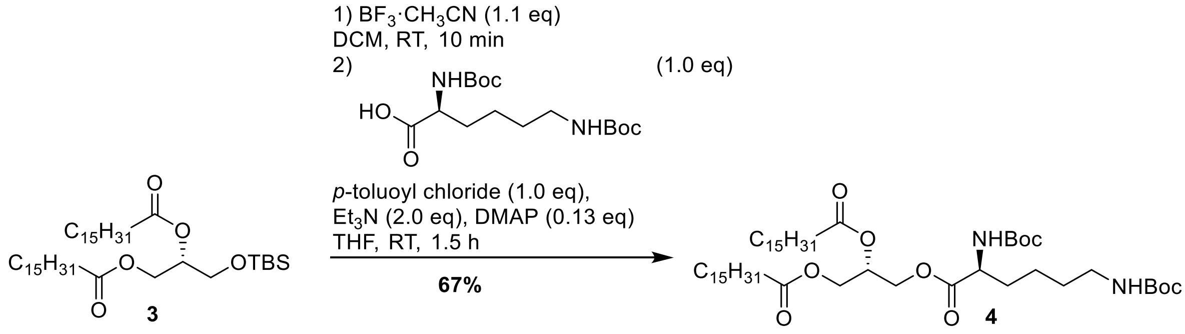

bioRxiv preprint doi: https://doi.org/10.1101/2025.11.06.685907; this version posted November 7, 2025. The copyright holder for this preprint (which was not certified by peer review) is the author/funder, who has granted bioRxiv a license to display the preprint in perpetuity. It is made available under aCC-BY 4.0 International license.

(*S*)-3-((N^2,N^6-bis(*tert*-butoxycarbonyl)-*L*-lysyl)oxy)propane-1,2-diyl-dipalmitate **4**

To an oven-dried flask, charged with TBS-protected diacylglyceride (1.04 g, 1.52 mmol) and DCM (15.2 mL) under nitrogen atmosphere, was added a 16 wt% solution of $BF_3 \cdot CH_3CN$ complex in CH_3CN (1.14 g, 1.67 mmol, 1.1 eq). The resulting yellow reaction was stirred at RT until completion after 10 min (TLC monitoring, pentane : Et_2O 98 : 2). The reaction was subsequently quenched by addition of chilled phosphate buffer (20 mL, 1 M, pH = 7) and diluted with Et_2O (20 mL). The resulting organic phase was washed with brine (20 mL), dried over $MgSO_4$ and concentrated *in vacuo* to yield the deprotected alcohol as a white solid (820 mg, 1.44 mmol, 95%).

This intermediate product was immediately redissolved in THF (15.2 mL), followed by addition of N^2,N^6-Bis-boc-*L*-lysine (52.8 mg, 1.52 mmol, 1.0 eq) and *p*-toluoyl chloride (200 µL, 1.51 mmol, 1.0 eq). After complete dissolution of the starting materials, Et_3N (0.42 mL, 3.02 mmol, 2.0 eq) and DMAP (23 mg, 0.19 mmol, 0.13 eq) were added, and an atmosphere of N_2 was established. The resulting reaction was stirred at RT until complete consumption of the amino-acid starting material (1.5 h, TLC monitoring, pentane : EtOAc : EtOH 6 : 3 : 1, ninhydrin stain). The reaction was acidified to pH 6 using 1 M aqueous HCl solution and partitioned between water (20 mL) and EtOAc (20 mL). The aqueous phase was extracted with EtOAc (2 x 20 mL). The combined organic phases were washed with sat. aqueous $NaHCO_3$ solution (30 mL), water (30 mL) and brine (30 mL), dried over $MgSO_4$ and concentrated *in vacuo*. The resulting red oil was dispersed on silica and purified using flash column chromatography on silica (pentane : Et_2O 80 : 20 to 70 : 30) to obtain **4** as a white wax (910 mg, 1.01 mmol, 67%).

^{1}H NMR (400 MHz, $CDCl_3$) δ 5.26 (p, *J* = 5.2 Hz, 1H), 5.07 (d, *J* = 8.3 Hz, 1H, (N-H)), 4.64 (s, 1H, (N-H)), 4.37 – 4.19 (m, 4H), 4.13 (dd, *J* = 11.9, 5.9 Hz, 1H), 3.11 (q, *J* = 6.5 Hz, 2H), 2.31 (td, *J* = 7.5, 2.1 Hz, 4H), 1.88 – 1.73 (m, 1H), 1.61 (dt, *J* = 13.2, 6.7 Hz, 7H), 1.55 – 1.46 (m, 1H), 1.46 – 1.40 (m, 18H), 1.40 – 1.34 (m, 2H), 1.25 (s, 48H), 0.92 – 0.84 (m, 6H).
^{13}C NMR (101 MHz, $cdcl_3$) δ 173.28, 172.89, 172.33, 68.67, 62.99, 61.89, 53.23, 39.96, 34.14, 34.03, 32.17, 31.92, 29.70, 29.65, 29.63, 29.50, 29.48, 29.36, 29.29, 29.27, 29.12, 29.09, 28.42, 28.31.
HRMS (ESI+) m/z calcd for $[M+Na]^+$ $C_{51}H_{96}N_2O_{10}Na$ = 919.6957; found 919.6944

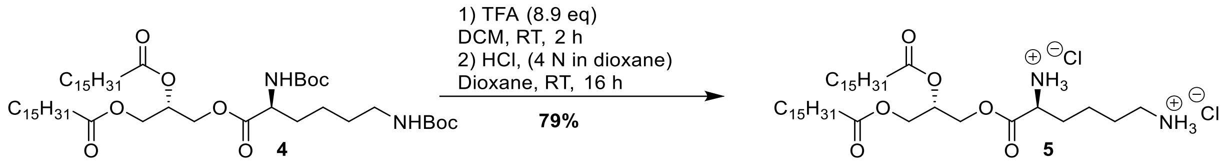

(*S*)-3-((*L*-lysyl)oxy)propane-1,2-diyl-dipalmitate bis hydrochloride salt **5** (Lys-DAG double chloride salt)

An oven-dried flask was charged with **4** (80 mg, 0.089 mmol), DCM (0.8 mL) and trifluoroacetic acid (0.2 mL, 0.80 mmol, 8.9 eq) under N_2 atmosphere. The resulting reaction was stirred at RT for 2 h (TLC monitoring, EtOAc : EtOH 4 : 1, ninhydrin stain). The reaction was then concentrated *in vacuo*, before redissolution in 1,4-dioxane (1.5 mL). To the resulting solution was added HCl (4 M solution in 1,4-dioxane, 89 µL). The reaction was stirred for 16 h at which point a white precipitate evidenced that the reaction had progressed. The reaction was concentrated *in vacuo*, redissolved in water and lyophilized to obtain **5** as a fine white powder (49 mg, 0.070 mmol, 79%).

bioRxiv preprint doi: https://doi.org/10.1101/2025.11.06.685907; this version posted November 7, 2025. The copyright holder for this preprint (which was not certified by peer review) is the author/funder, who has granted bioRxiv a license to display the preprint in perpetuity. It is made available under aCC-BY 4.0 International license.

^{1}H NMR (600 MHz, CD_3OD_SPE) δ 5.36 (tt, *J* = 6.4, 3.8 Hz, 1H), 4.48 (dd, *J* = 11.9, 4.2 Hz, 1H), 4.45 – 4.39 (m, 2H), 4.20 (dd, *J* = 12.1, 6.8 Hz, 1H), 4.12 (t, *J* = 6.5 Hz, 1H), 3.02 – 2.96 (m, 2H), 2.35 (dt, *J* = 10.1, 7.4 Hz, 4H), 2.06 – 1.89 (m, 2H), 1.75 (dq, *J* = 9.7, 7.7 Hz, 2H), 1.67 – 1.57 (m, 5H), 1.57 – 1.49 (m, 1H), 1.30 (d, *J* = 5.6 Hz, 48H), 0.91 (t, *J* = 7.0 Hz, 6H).

^{13}C NMR (151 MHz, MeOD) δ 174.81, 174.44, 170.17, 70.32, 65.40, 64.14, 63.25, 53.68, 49.57, 40.26, 35.03, 34.88, 33.09, 31.02, 30.83, 30.78, 30.69, 30.66, 30.50, 30.47, 30.23, 30.20, 28.07, 26.02, 23.75, 23.23, 14.45.

HRMS (ESI+) m/z calcd for $[M+H]^+$ $C_{41}H_{80}N_2O_6H$ = 697.6089; found 697.6084

Spectra:

bioRxiv preprint doi: https://doi.org/10.1101/2025.11.06.685907; this version posted November 7, 2025. The copyright holder for this preprint (which was not certified by peer review) is the author/funder, who has granted bioRxiv a license to display the preprint in perpetuity. It is made available under aCC-BY 4.0 International license.

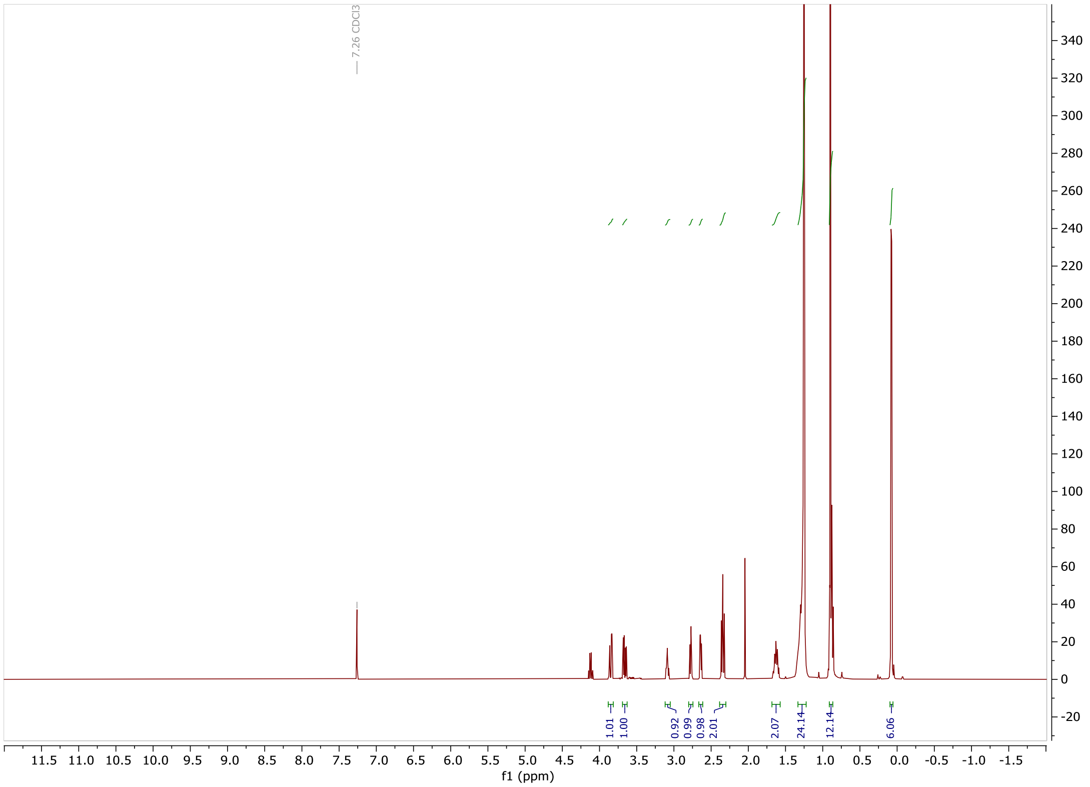

^{1}H-NMR of **2**

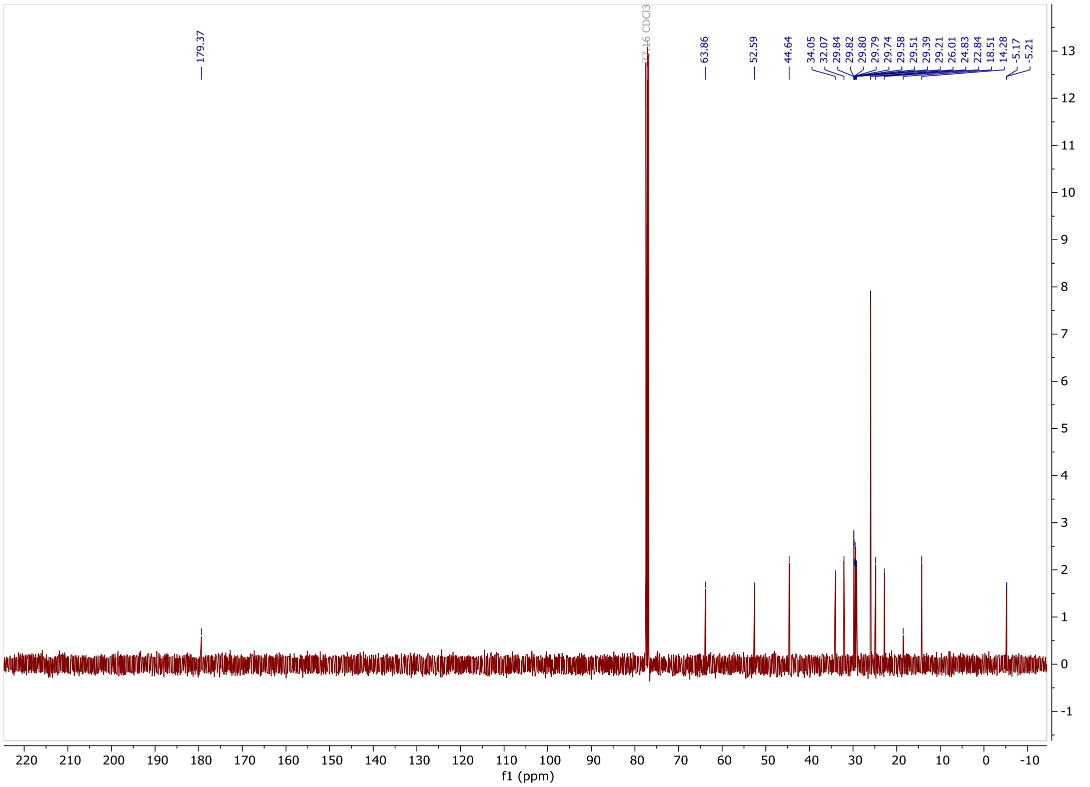

^{13}C-NMR of **2**

bioRxiv preprint doi: https://doi.org/10.1101/2025.11.06.685907; this version posted November 7, 2025. The copyright holder for this preprint (which was not certified by peer review) is the author/funder, who has granted bioRxiv a license to display the preprint in perpetuity. It is made available under aCC-BY 4.0 International license.

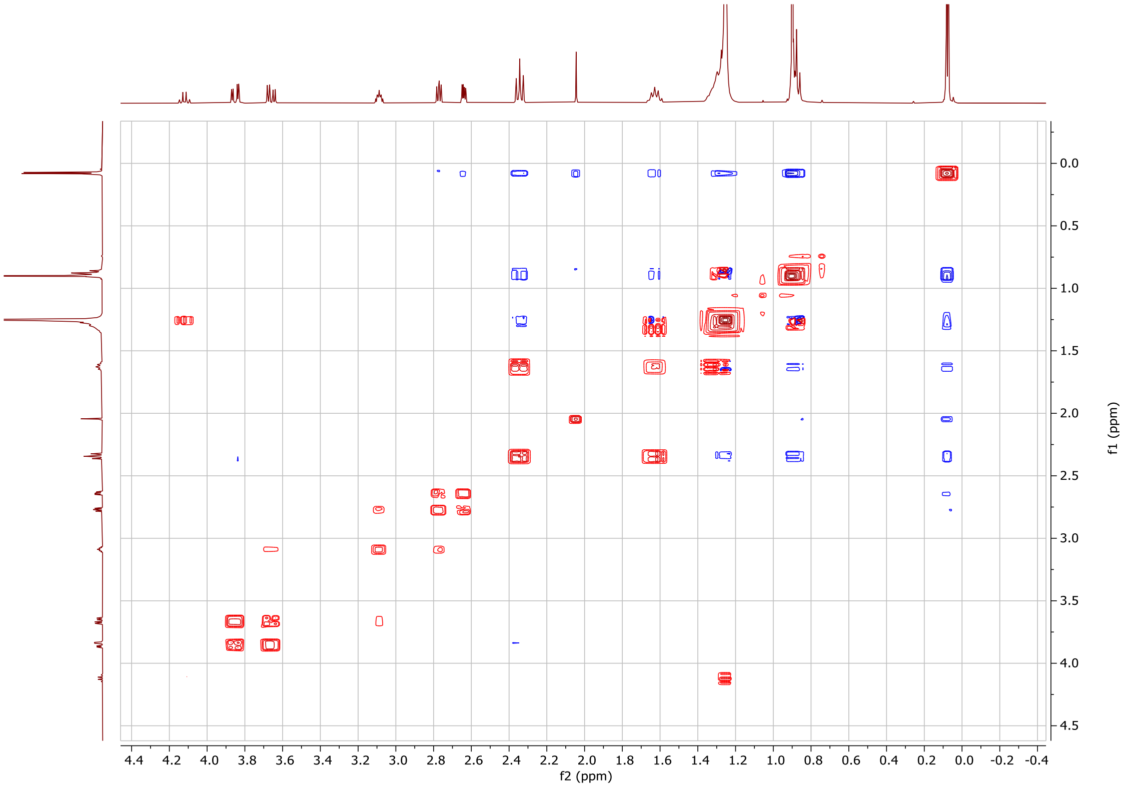

COSY of **2**

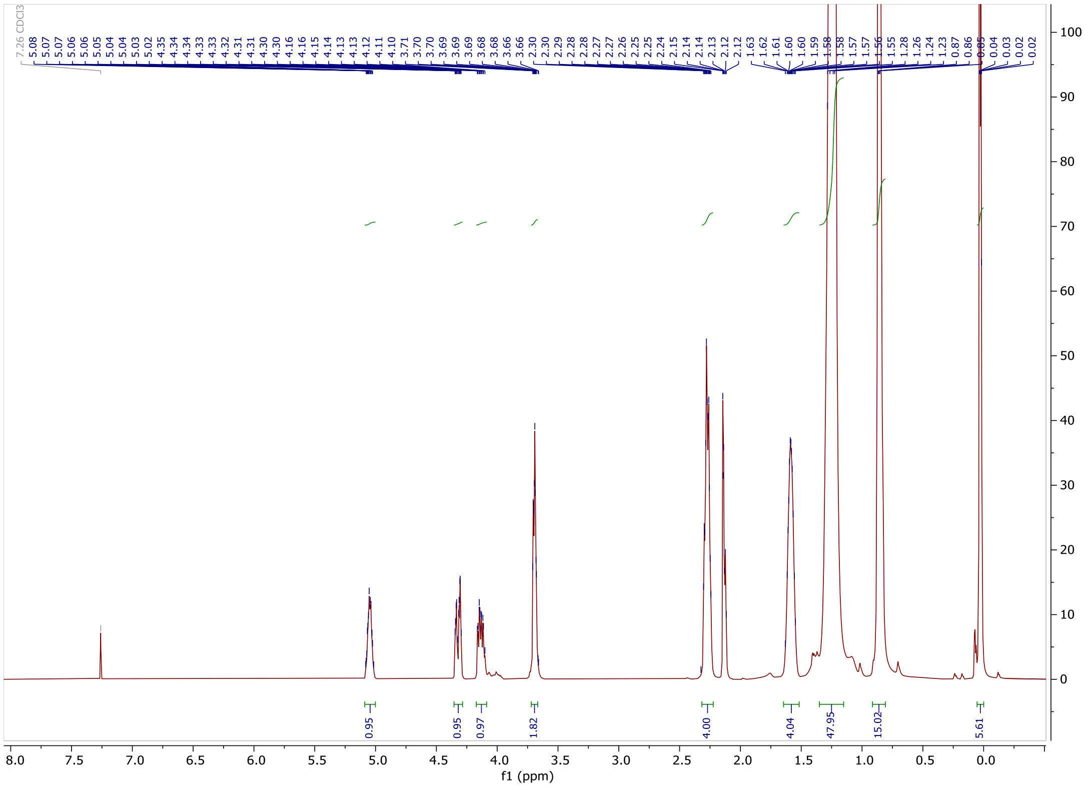

^{1}H-NMR of **3**

bioRxiv preprint doi: https://doi.org/10.1101/2025.11.06.685907; this version posted November 7, 2025. The copyright holder for this preprint (which was not certified by peer review) is the author/funder, who has granted bioRxiv a license to display the preprint in perpetuity. It is made available under aCC-BY 4.0 International license.

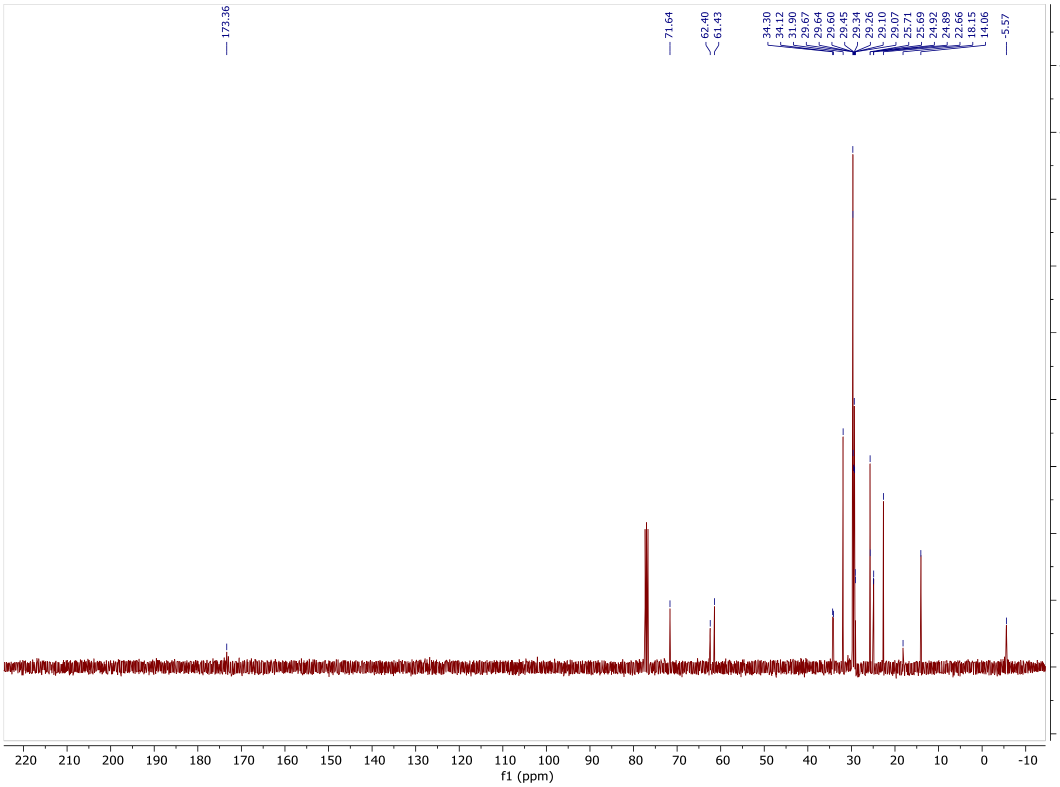

^{13}C-NMR of **3**

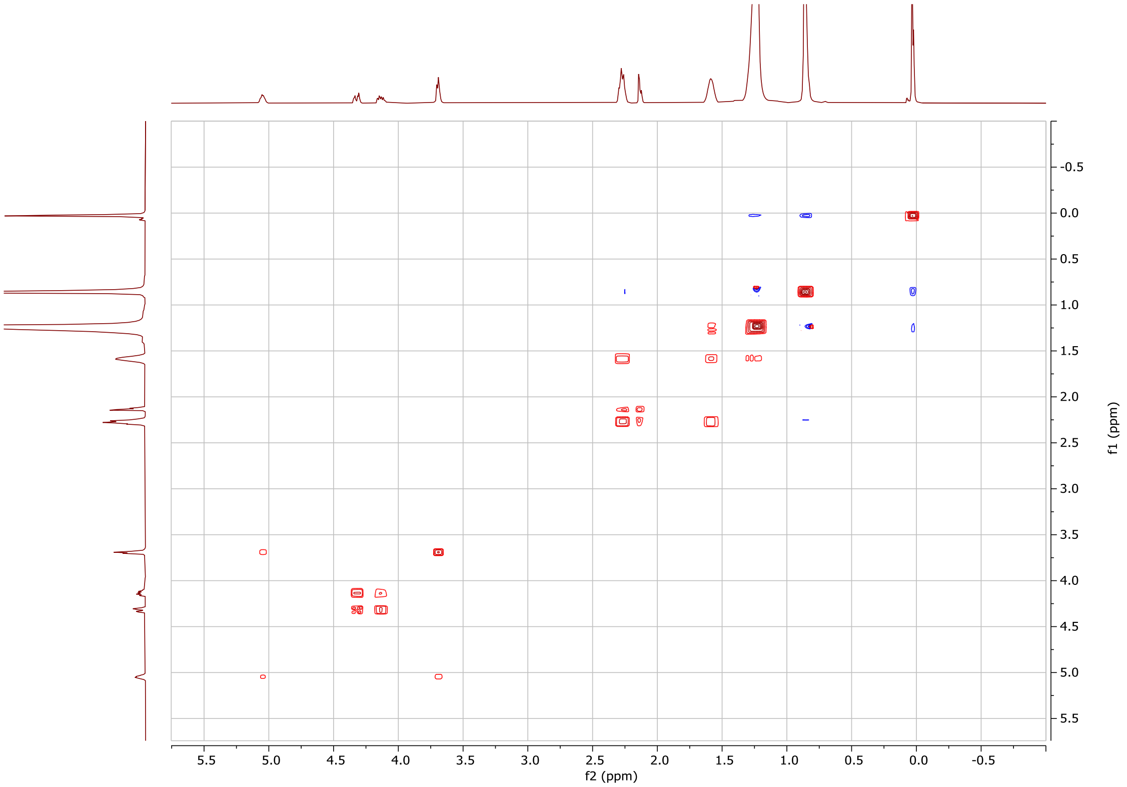

COSY of **3**

925

bioRxiv preprint doi: https://doi.org/10.1101/2025.11.06.685907; this version posted November 7, 2025. The copyright holder for this preprint (which was not certified by peer review) is the author/funder, who has granted bioRxiv a license to display the preprint in perpetuity. It is made available under aCC-BY 4.0 International license.

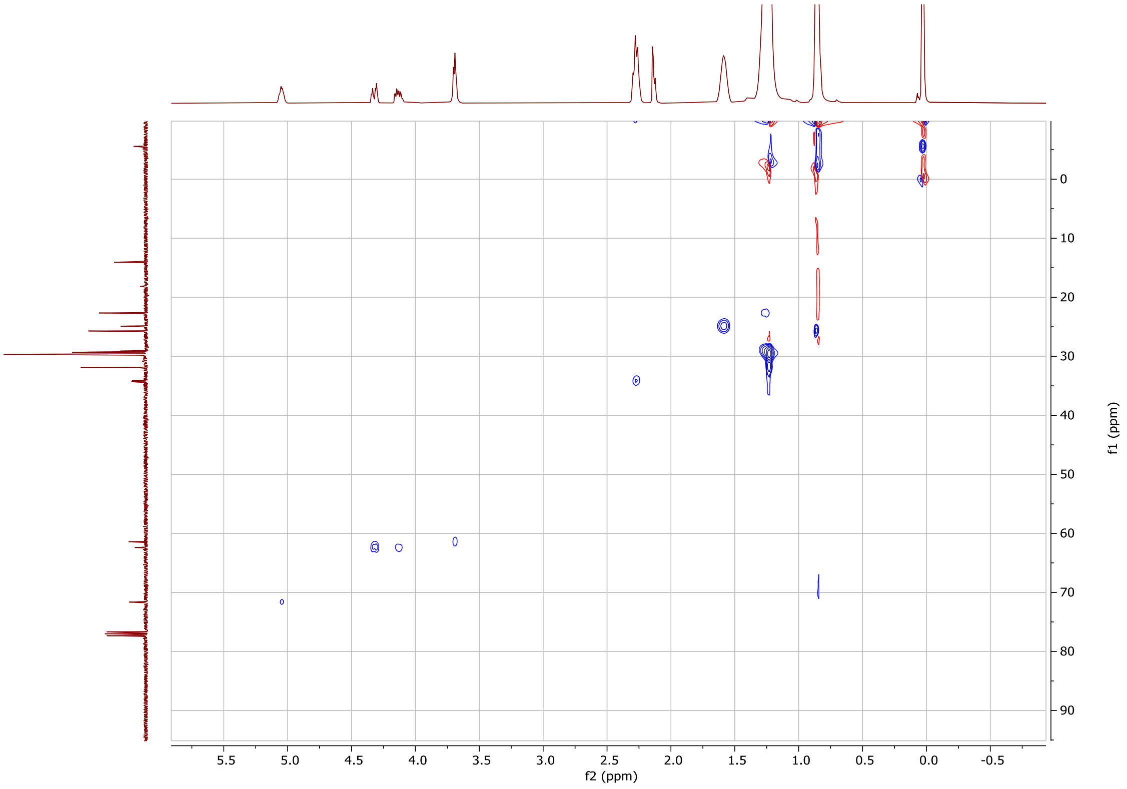

HSQC of **3**

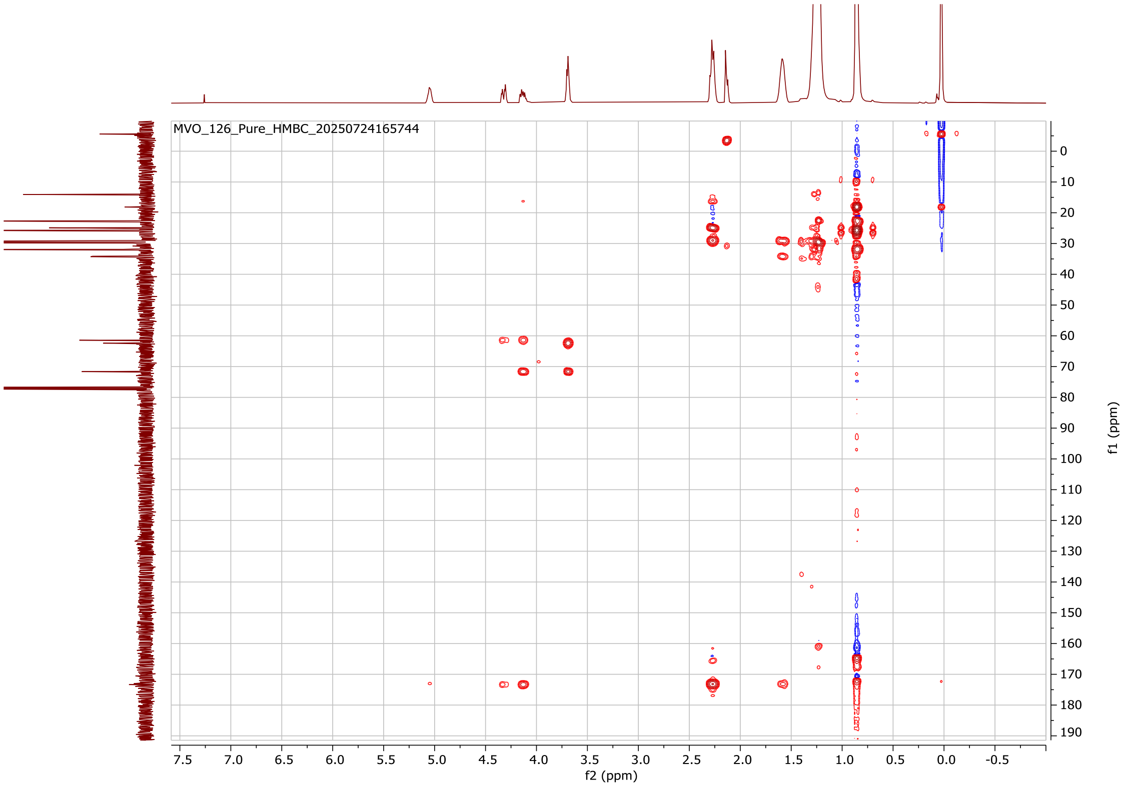

HMBC of **3**

926

bioRxiv preprint doi: https://doi.org/10.1101/2025.11.06.685907; this version posted November 7, 2025. The copyright holder for this preprint (which was not certified by peer review) is the author/funder, who has granted bioRxiv a license to display the preprint in perpetuity. It is made available under aCC-BY 4.0 International license.

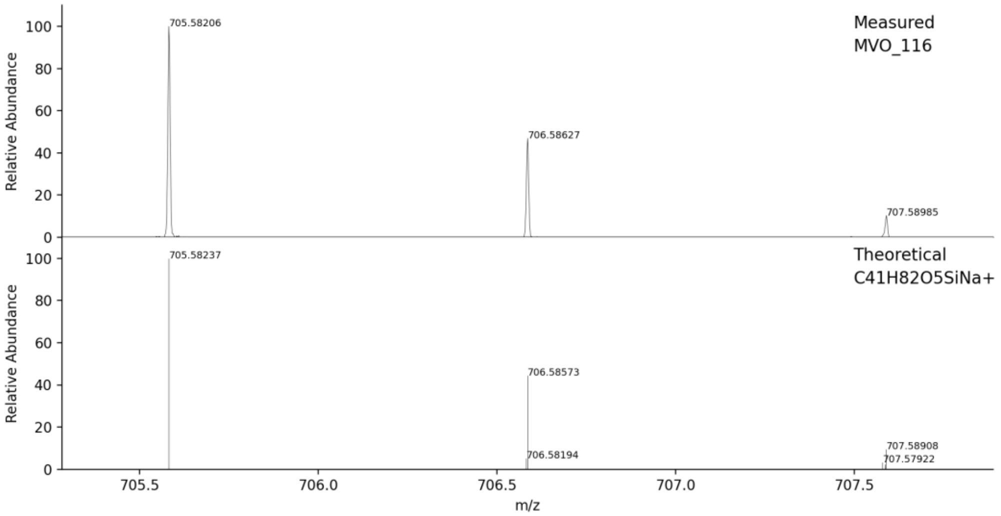

ESI-HRMS of **3**

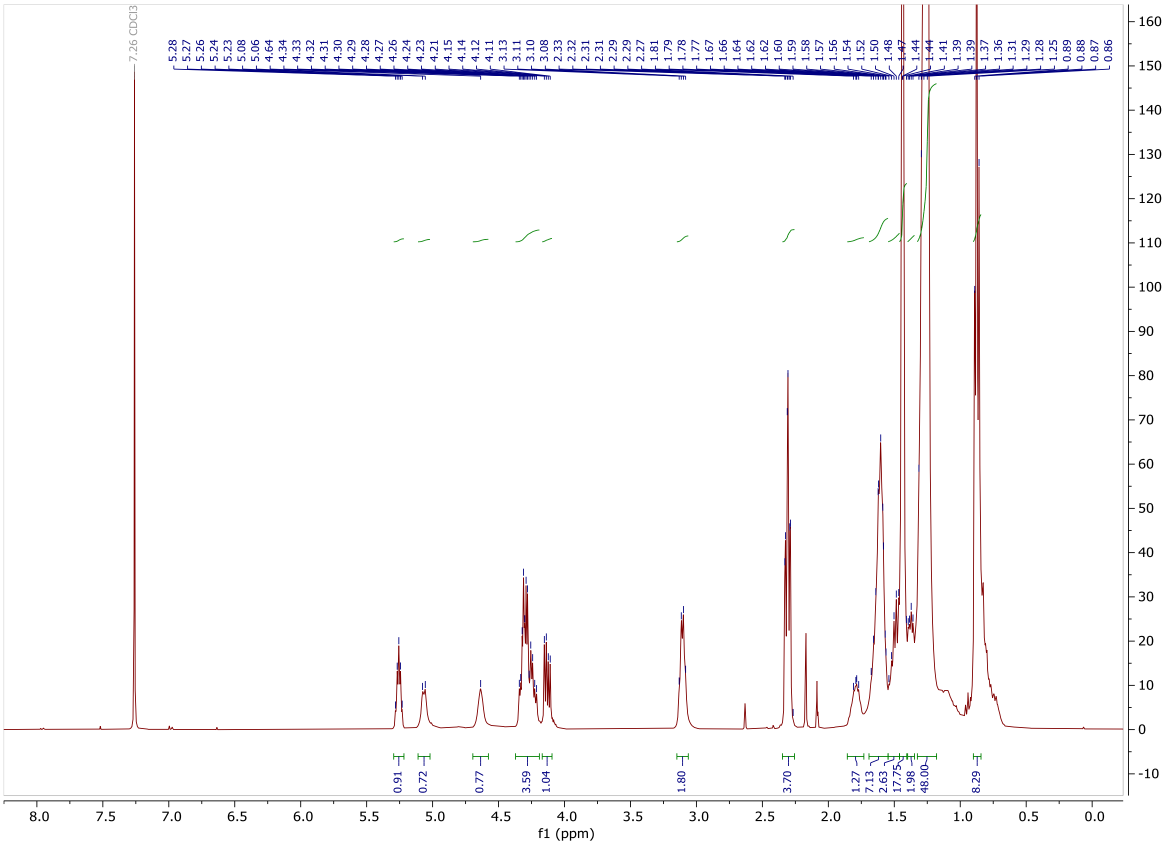

^{1}H-NMR of **4**

bioRxiv preprint doi: https://doi.org/10.1101/2025.11.06.685907; this version posted November 7, 2025. The copyright holder for this preprint (which was not certified by peer review) is the author/funder, who has granted bioRxiv a license to display the preprint in perpetuity. It is made available under aCC-BY 4.0 International license.

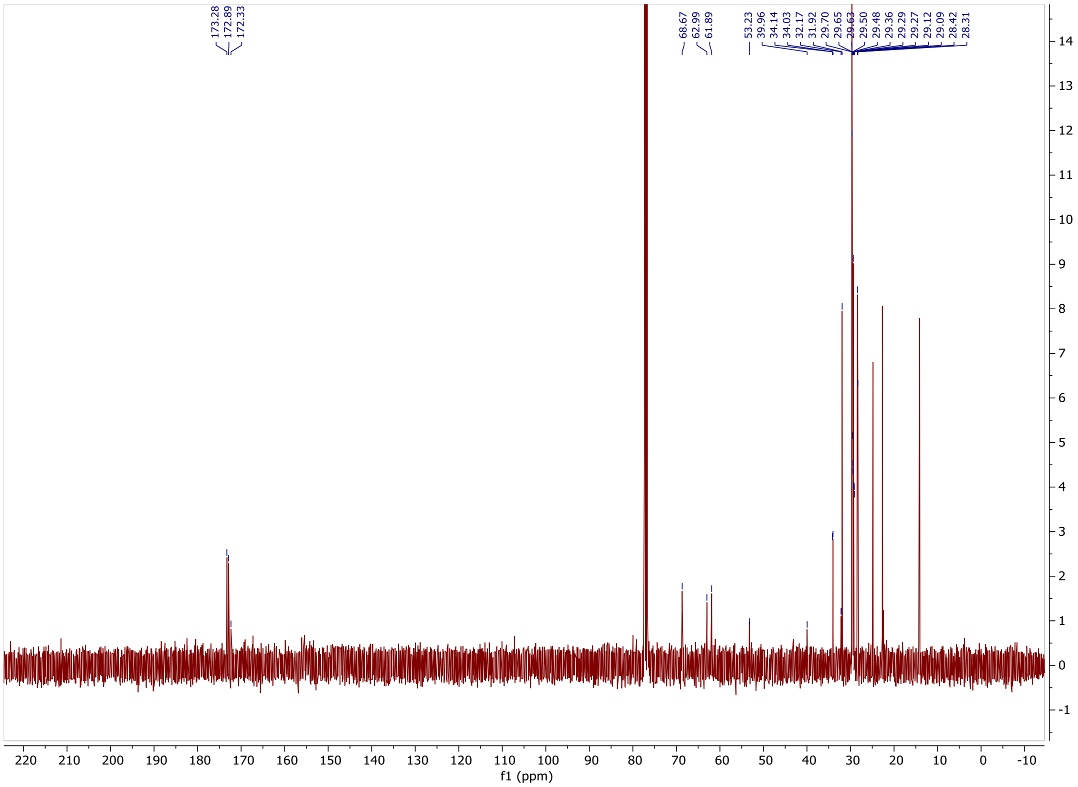

^{13}C-NMR of **4**

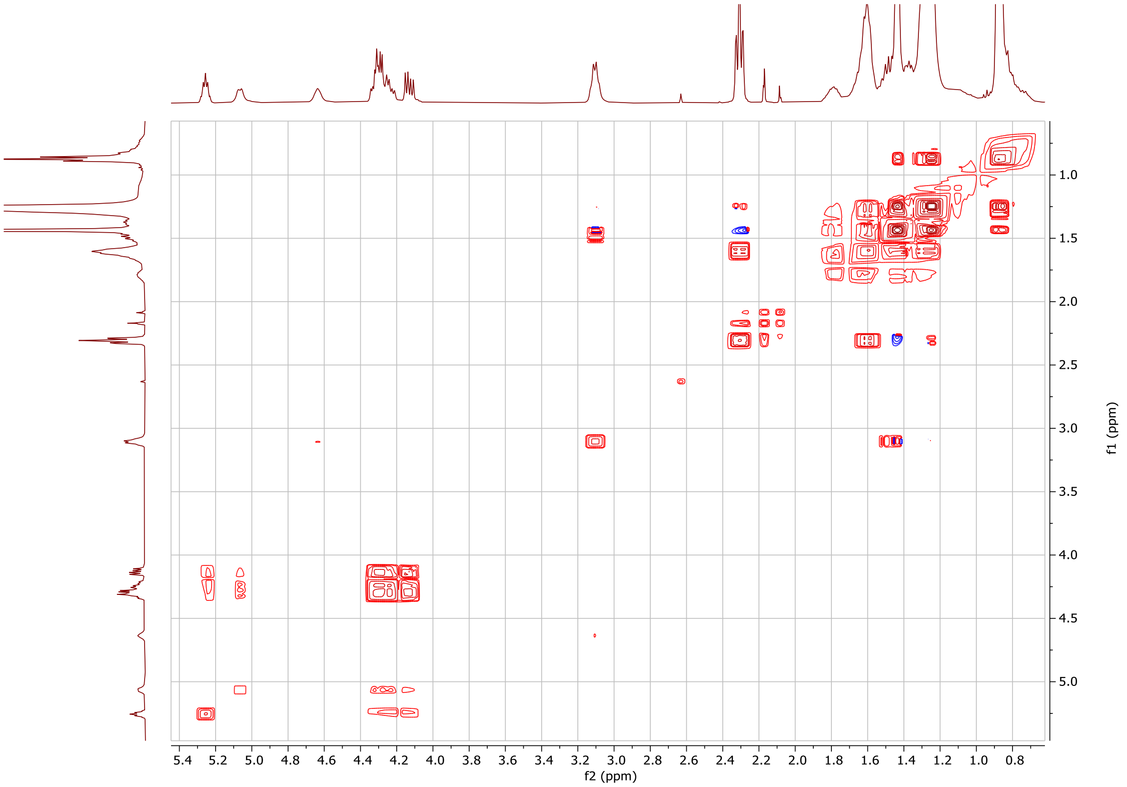

COSY of **4**

bioRxiv preprint doi: https://doi.org/10.1101/2025.11.06.685907; this version posted November 7, 2025. The copyright holder for this preprint (which was not certified by peer review) is the author/funder, who has granted bioRxiv a license to display the preprint in perpetuity. It is made available under aCC-BY 4.0 International license.

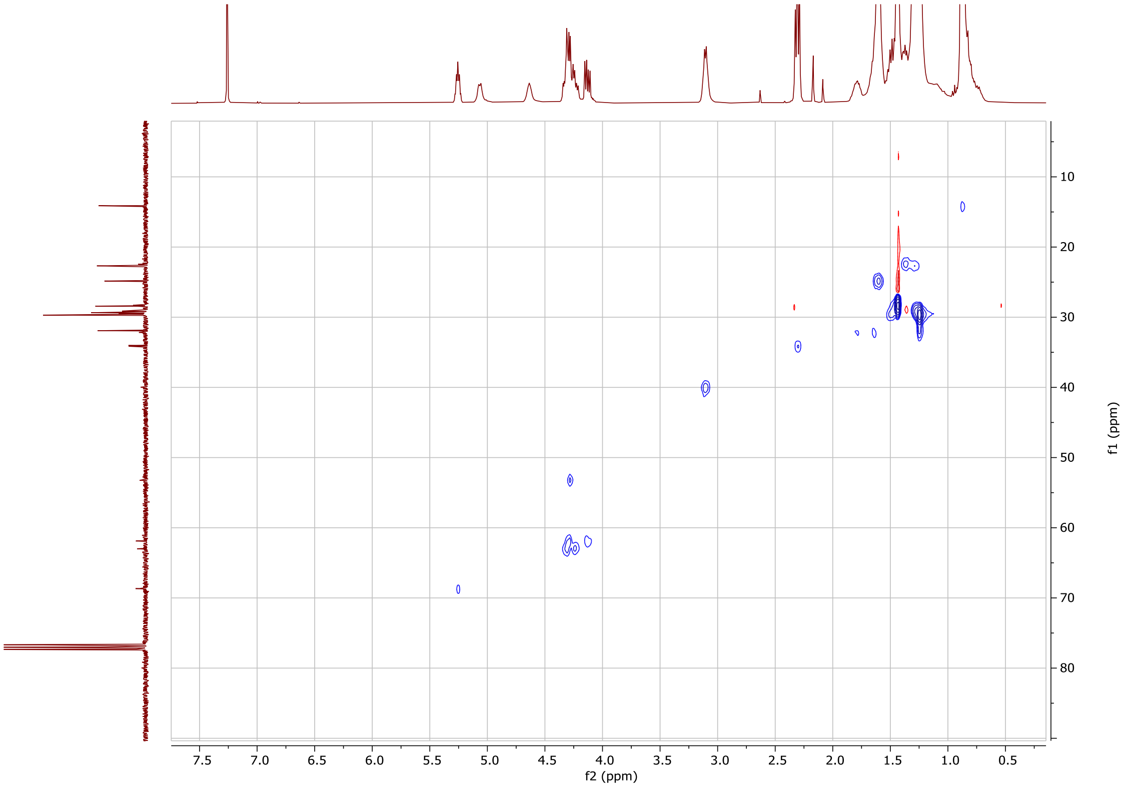

gHSQC of **4**

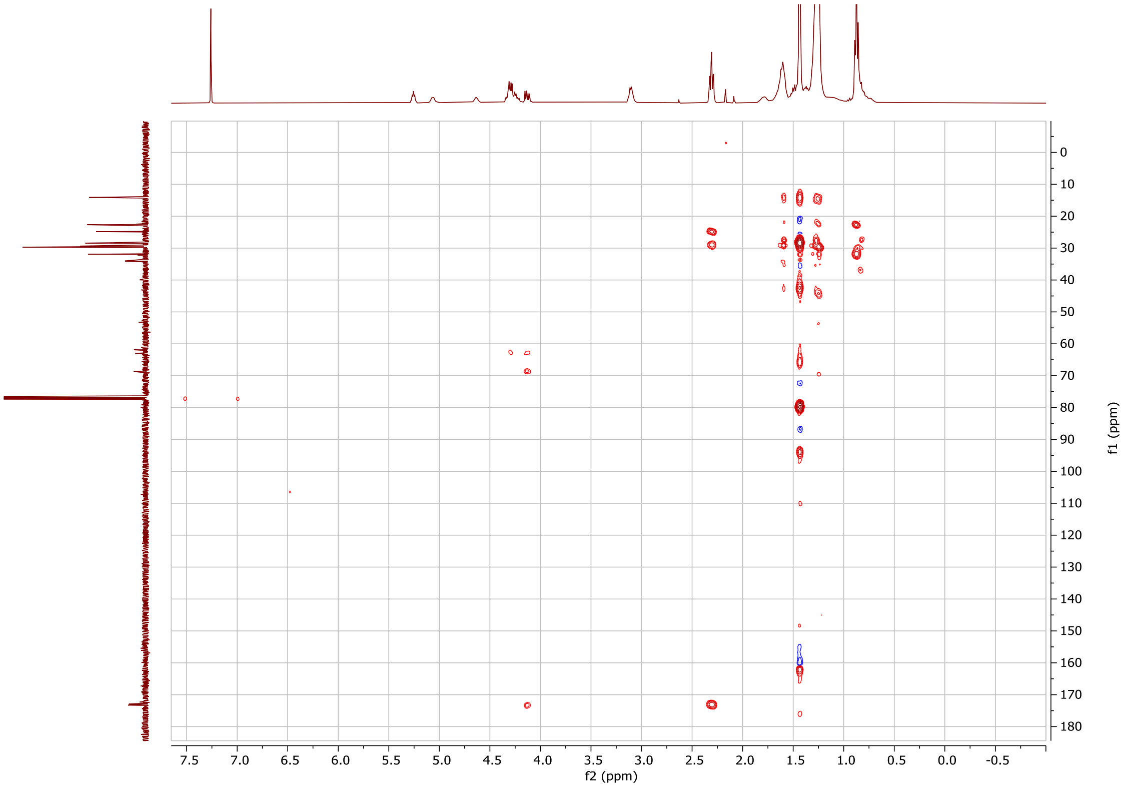

gHMBC of **4**

930

bioRxiv preprint doi: https://doi.org/10.1101/2025.11.06.685907; this version posted November 7, 2025. The copyright holder for this preprint (which was not certified by peer review) is the author/funder, who has granted bioRxiv a license to display the preprint in perpetuity. It is made available under aCC-BY 4.0 International license.

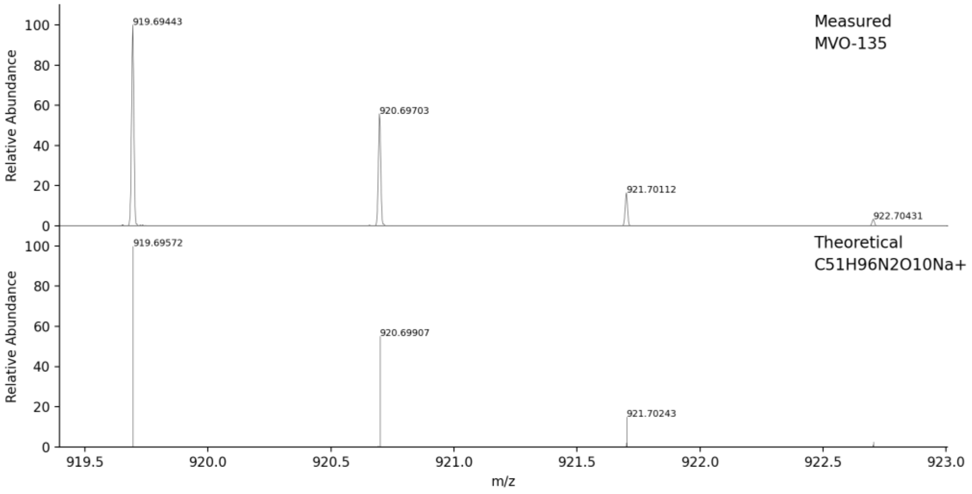

ESI-HRMS of **4**

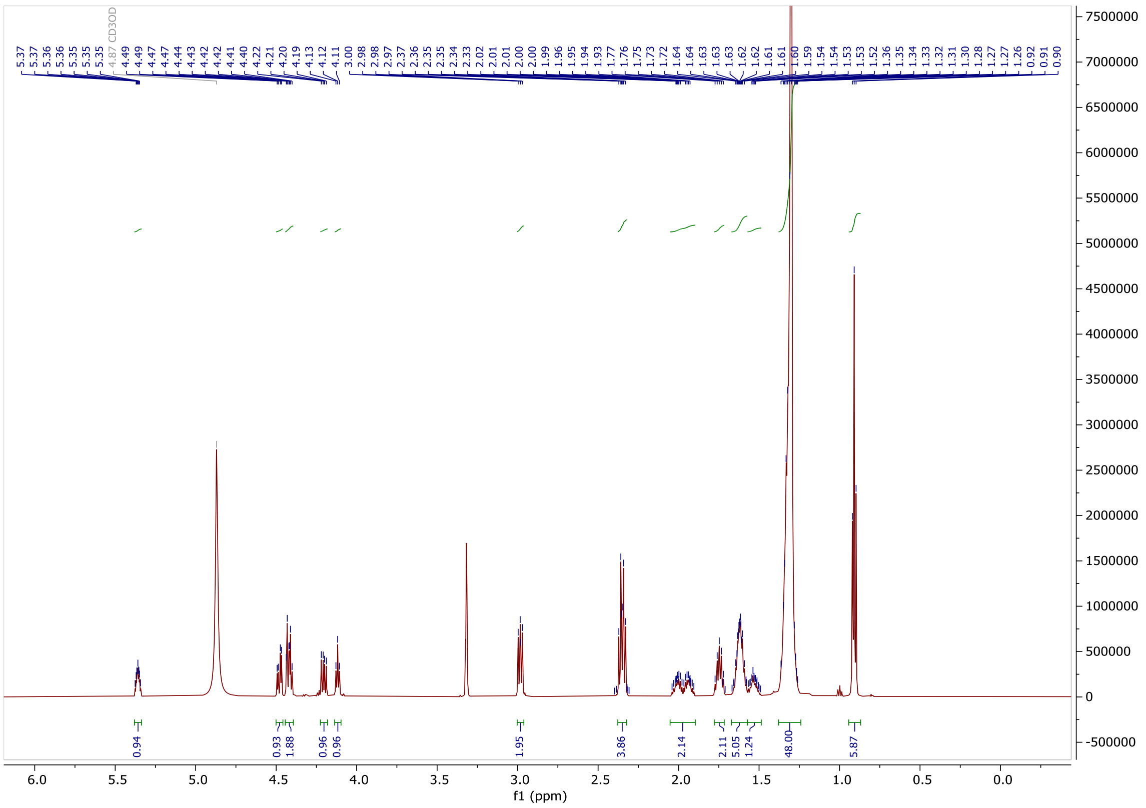

^{1}H-NMR of **5**

931

bioRxiv preprint doi: https://doi.org/10.1101/2025.11.06.685907; this version posted November 7, 2025. The copyright holder for this preprint (which was not certified by peer review) is the author/funder, who has granted bioRxiv a license to display the preprint in perpetuity. It is made available under aCC-BY 4.0 International license.

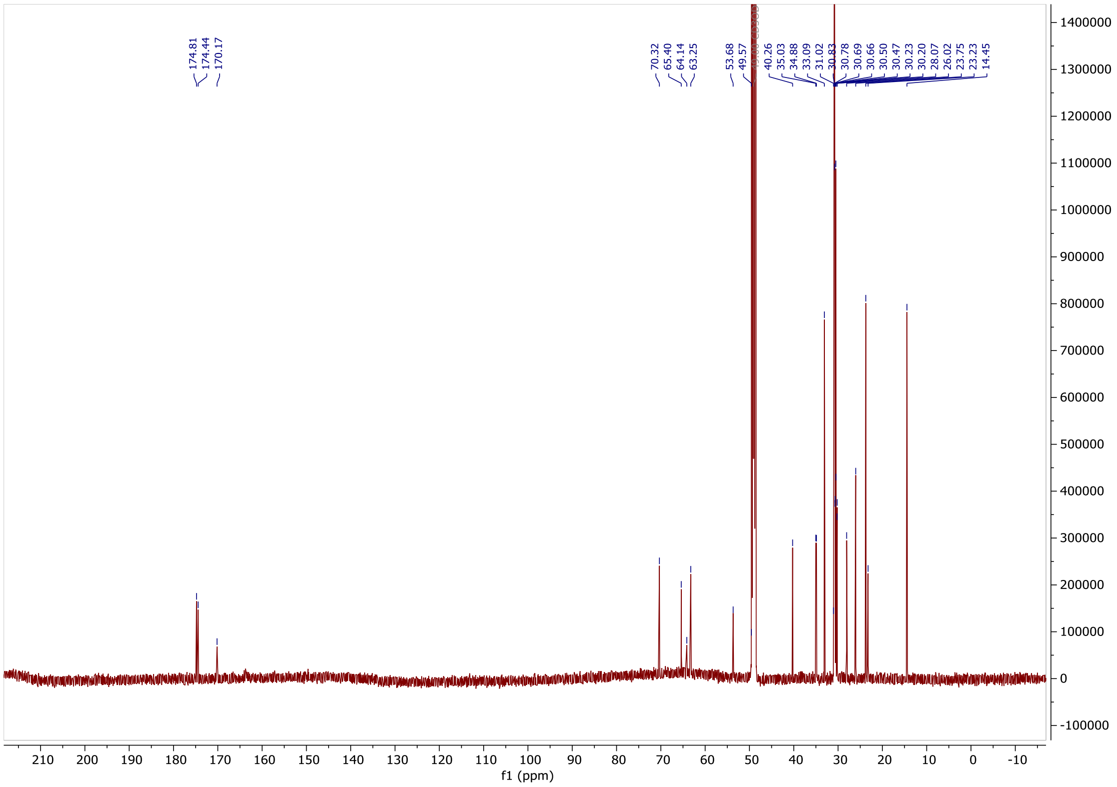

^{13}C-NMR of **5**

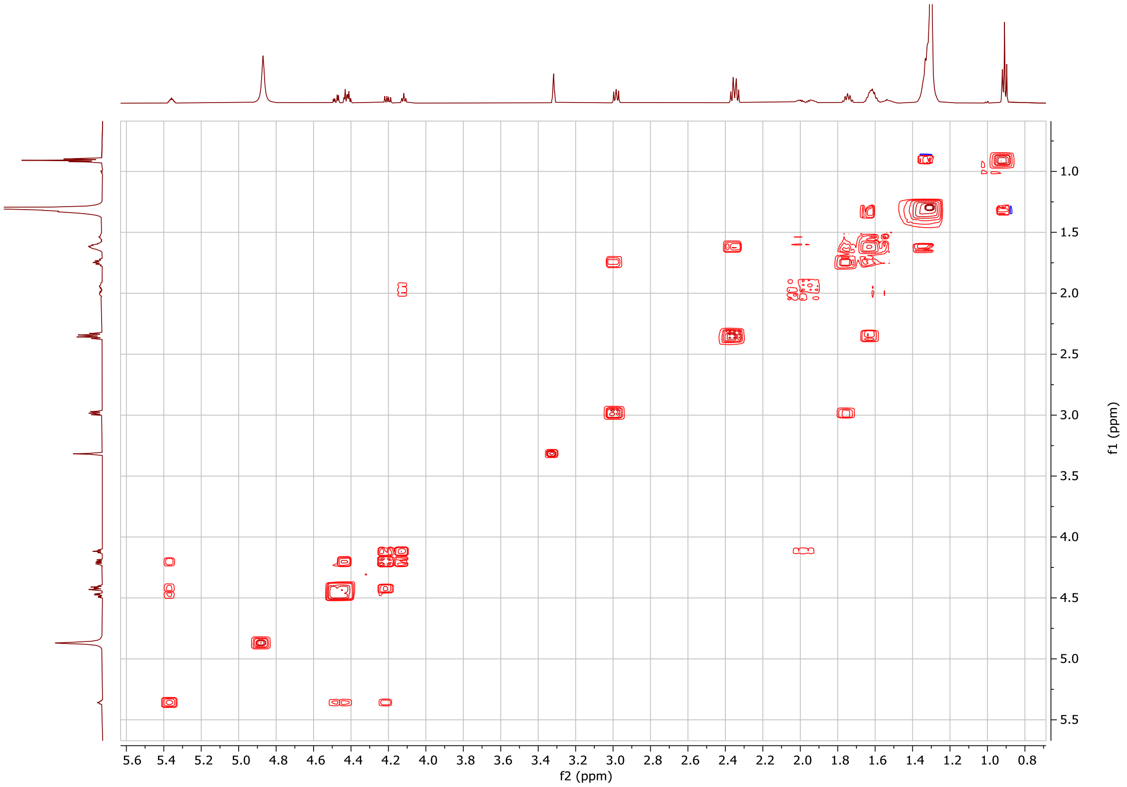

COSY of **5**

bioRxiv preprint doi: https://doi.org/10.1101/2025.11.06.685907; this version posted November 7, 2025. The copyright holder for this preprint (which was not certified by peer review) is the author/funder, who has granted bioRxiv a license to display the preprint in perpetuity. It is made available under aCC-BY 4.0 International license.

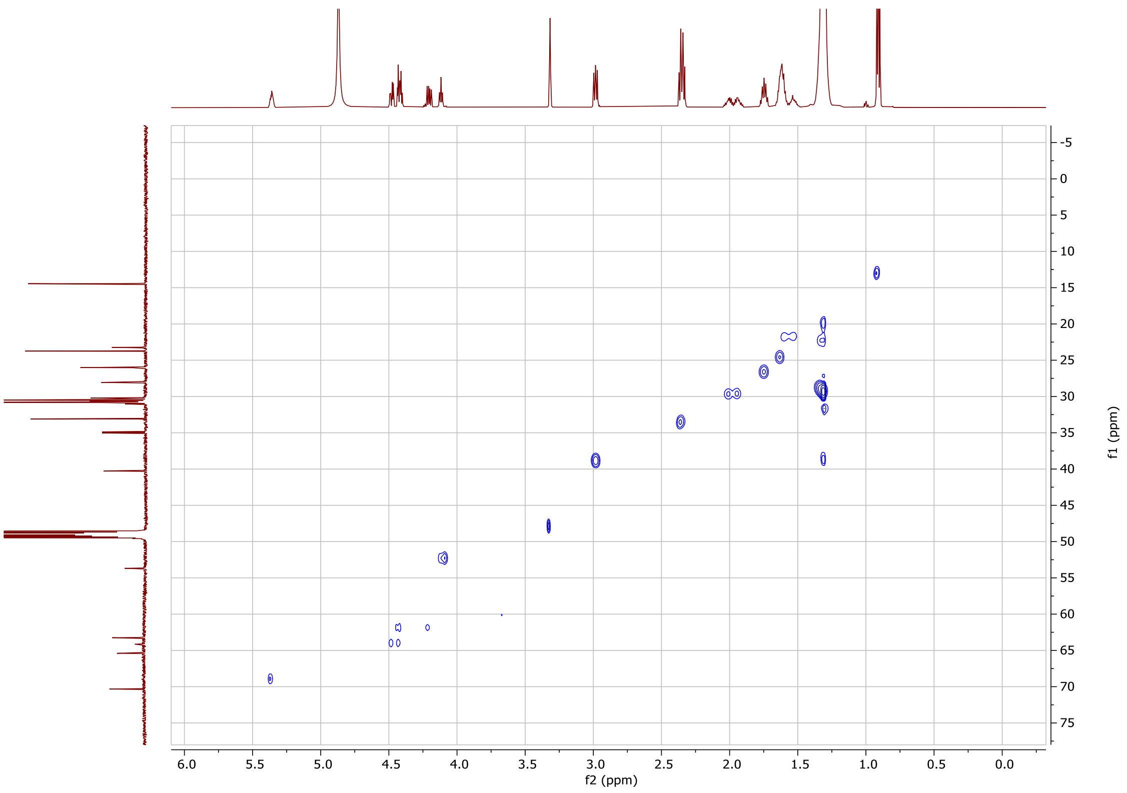

HSQC of **5**

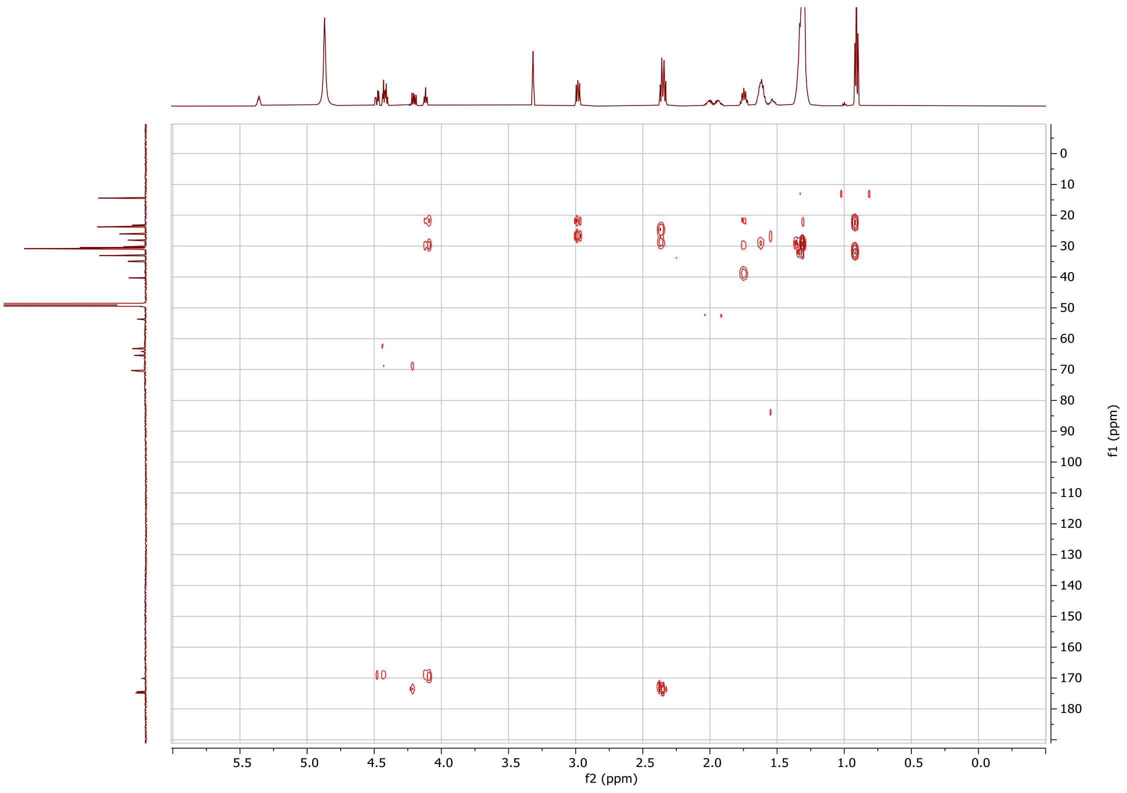

HMBC of **5**

933

bioRxiv preprint doi: https://doi.org/10.1101/2025.11.06.685907; this version posted November 7, 2025. The copyright holder for this preprint (which was not certified by peer review) is the author/funder, who has granted bioRxiv a license to display the preprint in perpetuity. It is made available under aCC-BY 4.0 International license.

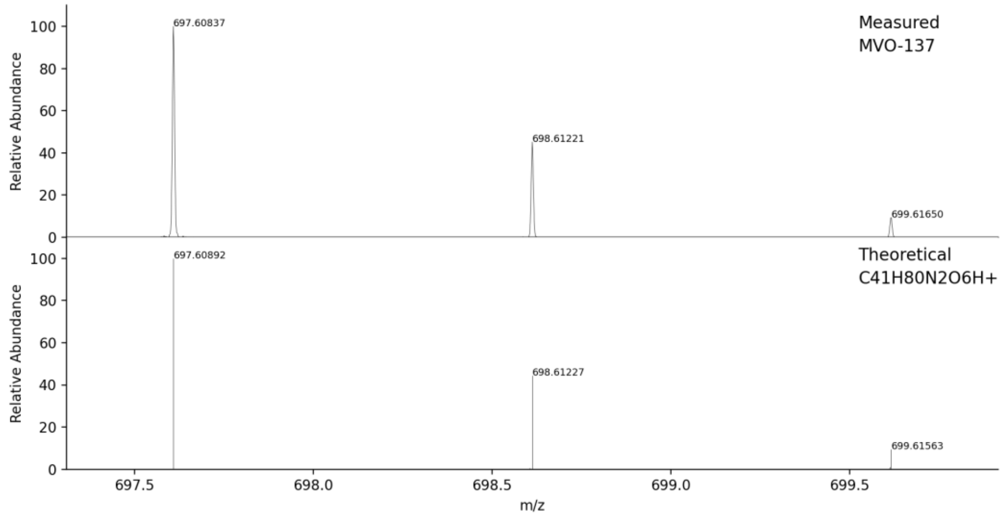

ESI-HRMS of **5**

Phylogeny of mycobacterial lysyldiacylglycerol production

Initial amino acid sequences for the generation of the phylogenetic tree of *lysX* and other aminoacyl transferases were obtained using Foldseek (*55*) of the Alphafold structure of MprF from *S. aureus* (Uniprot: Q2G2M2) and matching to the AFDB-SwissPROT database, yielding 152 sequences. An e-value for alignment threshold $< 1 \times 10^{-12}$ reduced the number of matching sequences to 41. After adding 10 sequences in bacteria of interest identified using Blastp, a final 51 sequences were aligned using Clustal Omega (*56*). The evolutionary analyses were conducted by the Maximum Likelihood Method in MEGA12 (*57*) using up to 6 parallel computing threads to identify the tree with the highest log likelihood (-56 974.80), and visualized using FigTree (available at: http://tree.bio.ed.ac.uk/software/figtree).

M. marinum lysX mutant and complement

Disruption of the *lysX*/MMAR_2447 gene in *M. marinum* was confirmed through isolation and sequencing of a strain with a transposon insertion in MMAR_2447, 10.6% of the way through the *LysX* open reading frame, from a sequenced arrayed transposon library (kindly provided by C. Cosma and L. Ramakrishnan, University of Cambridge UK). Both the wildtype strain and transposon mutant were then transformed with *msp12:cerulean,* a plasmid constitutively expressing the fluorescent protein cerulean under the *msp12* promoter. For complementation, the entire *M. marinum lysX* open reading frame was cloned behind the *hsp60* promoter and inserted into the *msp12:cerulean* plasmid.

Zebrafish husbandry and *M. marinum* infections

Zebrafish at two days post-fertilization were infected via the caudal vein as described (*58*) with 150–200 fluorescent bacteria per fish. All experiments had a minimum of 30 individuals per group on at least three different days to provide biologically and statistically robust data. Infection burden was measured at one day post-infection and four days post-infection through measurement of fluorescence. Fluorescence is enumerated by calculating the number of pixels above background using a constant threshold. Results were analyzed using GraphPad Prism, using Welch's ANOVA followed by a Dunnett's T3 multiple comparison

bioRxiv preprint doi: https://doi.org/10.1101/2025.11.06.685907; this version posted November 7, 2025. The copyright holder for this preprint (which was not certified by peer review) is the author/funder, who has granted bioRxiv a license to display the preprint in perpetuity. It is made available under aCC-BY 4.0 International license.

of each group to the WT strain. Representative images of each group were chosen from the median values. Zebrafish husbandry and experimental procedures were performed in accordance and compliance with policies approved by the Duke University Institutional Animal Care and Use Committee (protocol A049-23-03).

bioRxiv preprint doi: https://doi.org/10.1101/2025.11.06.685907; this version posted November 7, 2025. The copyright holder for this preprint (which was not certified by peer review) is the author/funder, who has granted bioRxiv a license to display the preprint in perpetuity. It is made available under aCC-BY 4.0 International license.

Figure S1 Nair *et al.* 2025

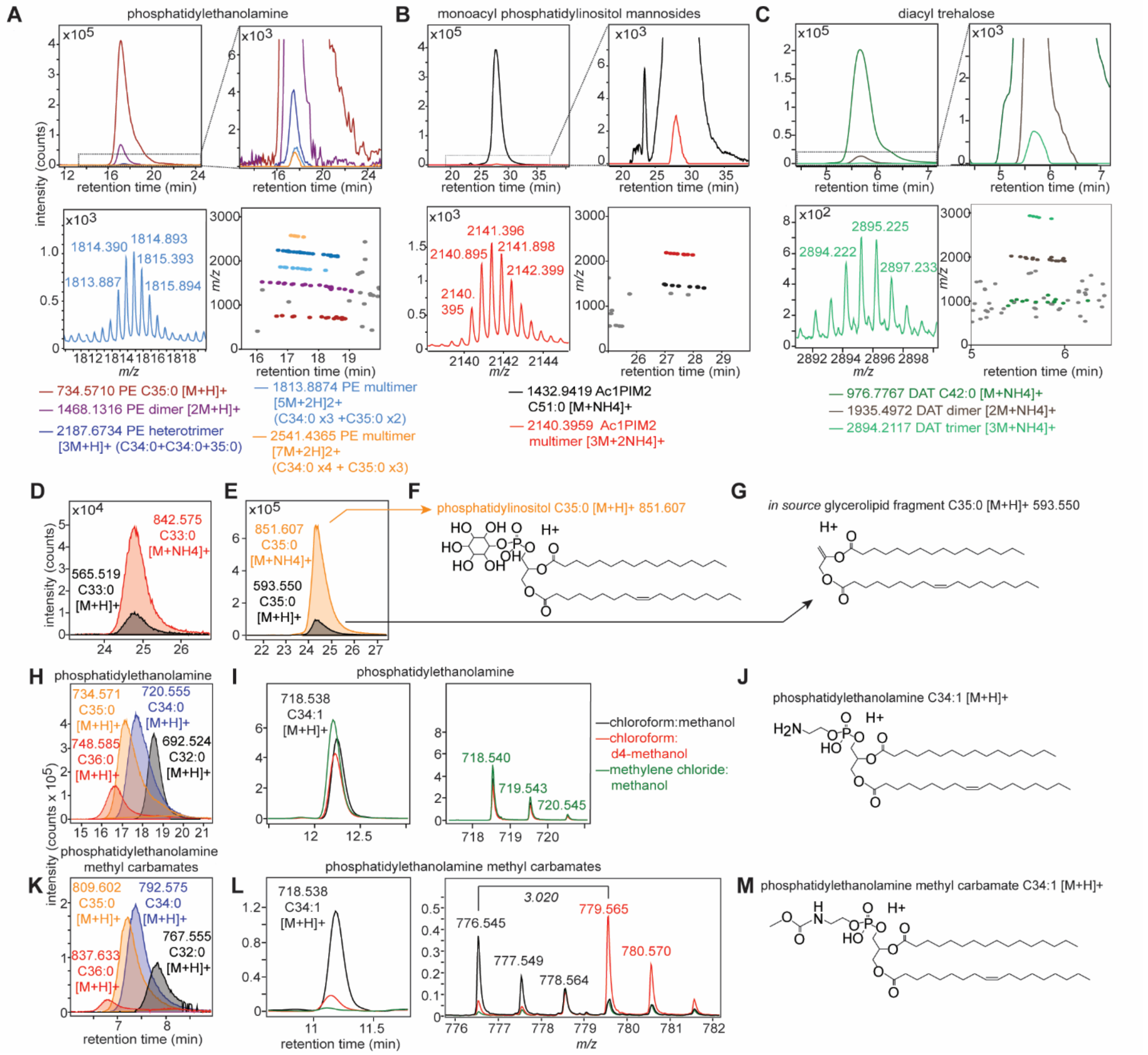

Fig. S1. Identification of *in source* multimers, fragments and solvent-induced modification of known Mtb lipids. (**A**) phosphatidyl ethanolamine (PE), (**B**) monoacyl phosphatidylinositol dimannoside (Ac1PIM2) and (**C**) diacyl trehalose (DAT) multimer chromatograms were censored manually in the credentialling pipeline (Fig. 1F). The inset for each panel expands low-intensity multimer peaks, *top right*; representative mass spectra of multimer adducts, *bottom left*; and mapping the identified multimers onto retention time space, *bottom right*. (**D** to **G**) Representative (**D** and **E**) chromatograms and (**F**) structure of phosphatidylinositol (PI) acylforms with overlapping peaks that were consistent with (**G**) glycerolipid fragments (*black*). (**H**) Representative chromatograms of acylform distributions of PE in *M. marinum.* (**I**) Chromatograms, *left*, mass spectra, *right*, and (**J**) a representative structure show consistent detection of PE with modified solvents including deuterated methanol and methylene chloride for extraction. (**K**) Representative chromatograms of acylform distributions of PE methyl carbamates in *M. marinum*. (**L**) Chromatograms, *left*, mass spectra, *right*, and (**M**) a representative structure show loss of PE methyl carbamates with methylene chloride extraction and d4-methanol integration demonstrated by a mass shift of 3.020.

bioRxiv preprint doi: https://doi.org/10.1101/2025.11.06.685907; this version posted November 7, 2025. The copyright holder for this preprint (which was not certified by peer review) is the author/funder, who has granted bioRxiv a license to display the preprint in perpetuity. It is made available under aCC-BY 4.0 International license.

Figure S2 Nair *et al.* 2025

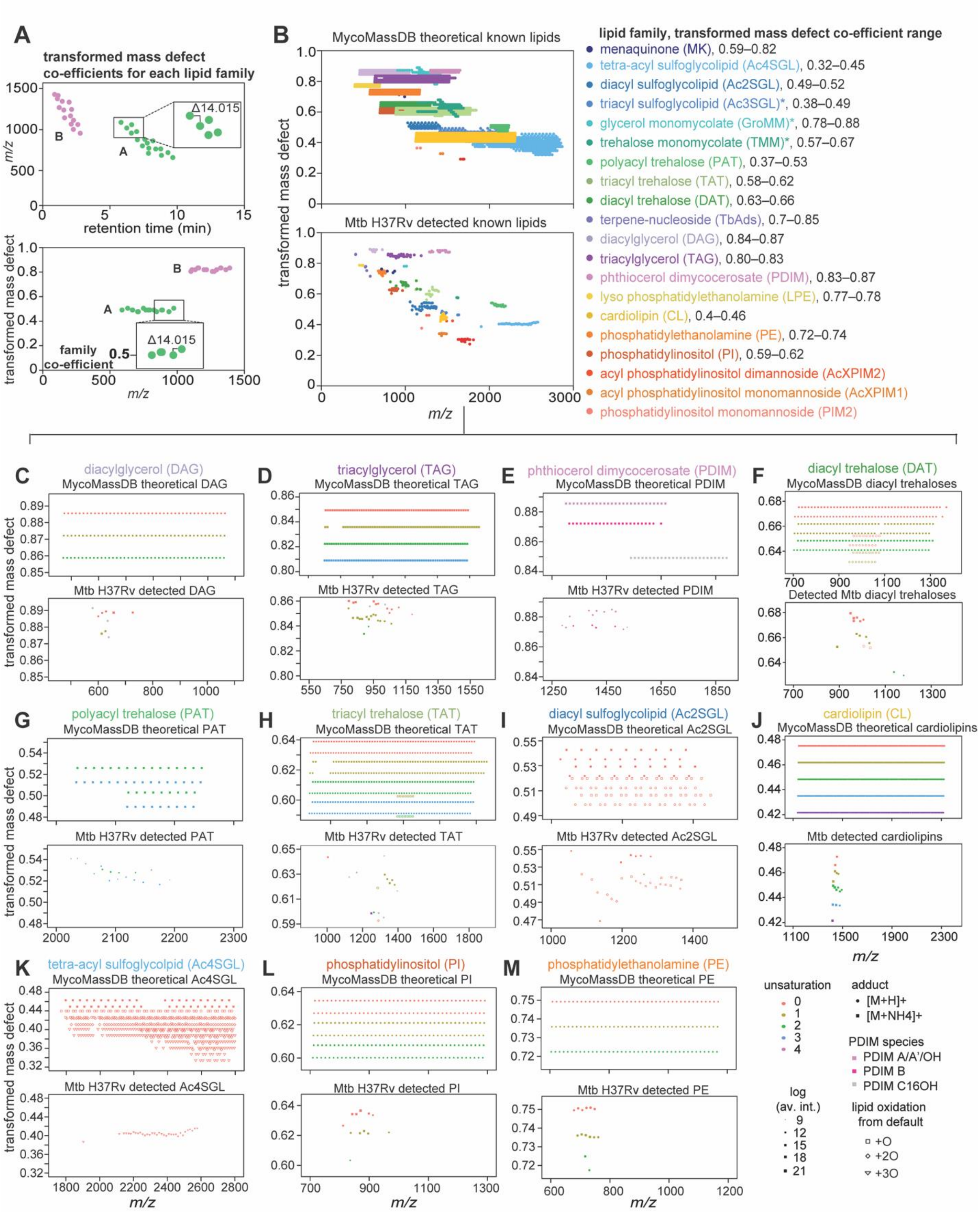

Fig. S2. A methylene transformed mass defect of known *M. tuberculosis* lipids. (**A**) Schematic of example theoretical lipid families represented as *m/z* vs retention time, *top*, and as transformed or Kendrick mass defect vs *m/z*, *bottom*, to identify co-efficients for each lipid family. (**B**) Theoretical known lipids from

bioRxiv preprint doi: https://doi.org/10.1101/2025.11.06.685907; this version posted November 7, 2025. The copyright holder for this preprint (which was not certified by peer review) is the author/funder, who has granted bioRxiv a license to display the preprint in perpetuity. It is made available under aCC-BY 4.0 International license.

MycoMassDB were transformed into a scale where related acylforms aligned along a horizontal axis, *top*. The detectable lipids from the credentialed Mtb H37Rv lipidome as in Figure 1I were also transformed showing narrower acylform distributions, *bottom*. The range of transformed mass defects of lipid families from MycoMassDB is appended to the legend. For each lipid family with alkylforms where a lead compound was confirmed by collisional MS a focused plot as in Fig. S2*A* shows the distribution of theoretical and detectable alkylforms, where differences in unsaturation, oxygenation, and ionization adduct are observed as horizontal displacements in (**C**) diacylglycerol, (**D**) triacylglycerol, (**E**) phthiocerol dimycocerosate, (**F**) diacyl trehalose, (**G**) polyacyltrehalose, (**H**) triacyl trehalose, (**I**) diacyl sulfoglycolipid, (**J**) cardiolipin, (**K**) tetra-acyl sulfoglycolipid, (**L**) phosphatidyl inositol, and (**M**) phosphatidyl ethanolamine. Point size was scaled to the log of average intensity across the matched quadruplicate cultures of Mtb H37Rv analyzed. Lipids with asterisks mass matched to MycoMassDB and were not studied by CID-MS.

bioRxiv preprint doi: https://doi.org/10.1101/2025.11.06.685907; this version posted November 7, 2025. The copyright holder for this preprint (which was not certified by peer review) is the author/funder, who has granted bioRxiv a license to display the preprint in perpetuity. It is made available under aCC-BY 4.0 International license.

Figure S3 (1)

Nair *et al.* 2025

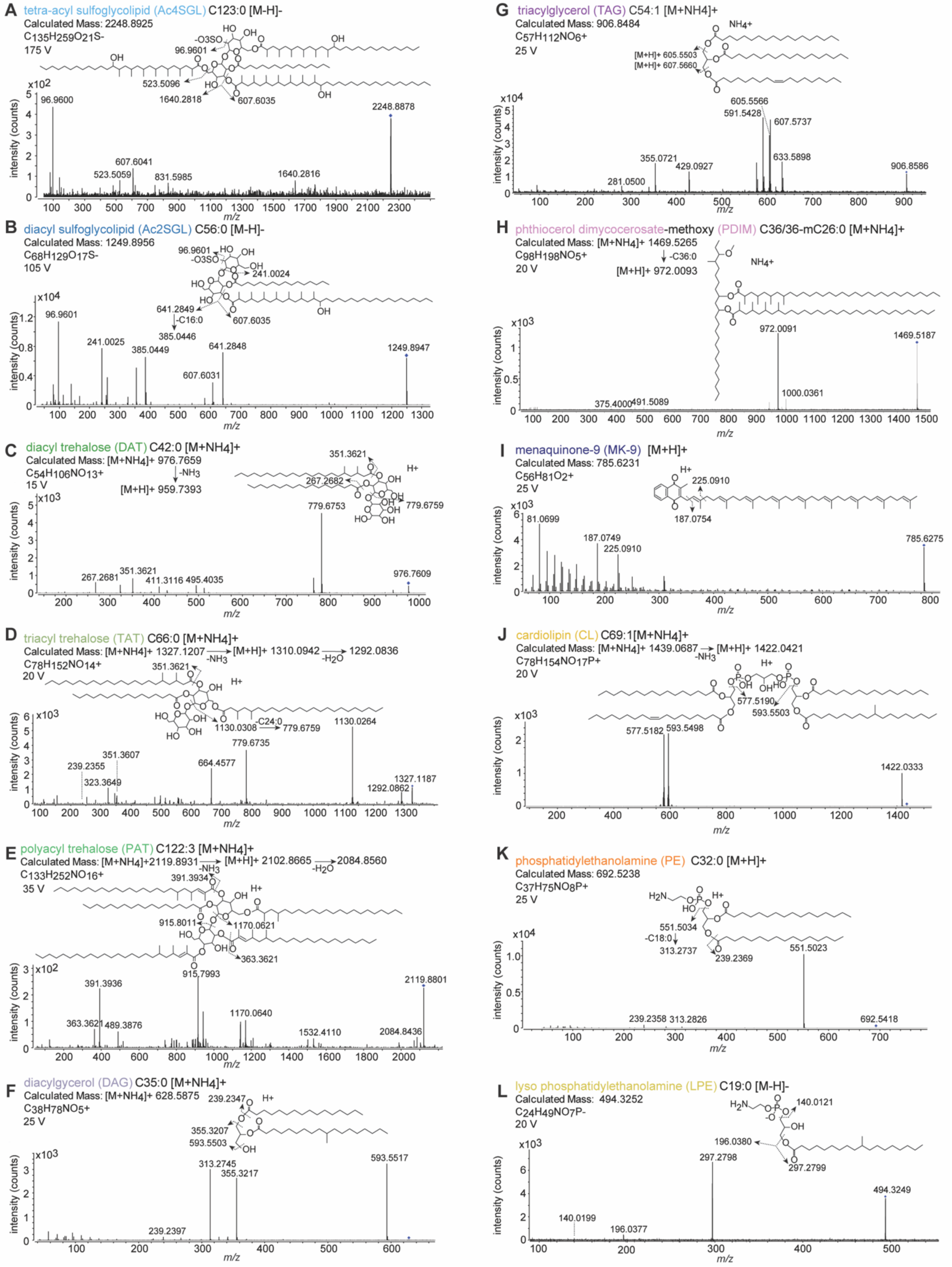

bioRxiv preprint doi: https://doi.org/10.1101/2025.11.06.685907; this version posted November 7, 2025. The copyright holder for this preprint (which was not certified by peer review) is the author/funder, who has granted bioRxiv a license to display the preprint in perpetuity. It is made available under aCC-BY 4.0 International license.

Figure S3 (2) Nair *et al.* 2025

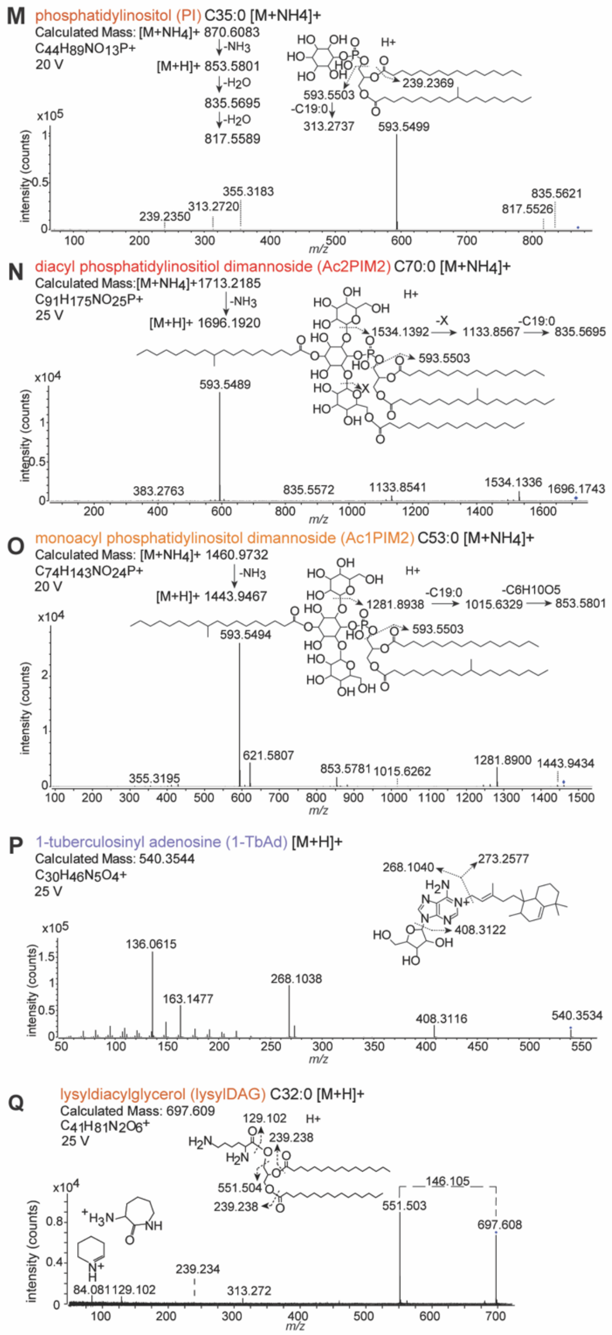

Fig. S3. Collisional MS spectra of *M. tuberculosis* lipids. Collisional MS was performed on lead compounds from the 'lipidome of knowns' in either the negative or positive modes with a voltage optimized to each compound. A representative chemical structure and overlay of fragments detected is shown for (**A**) tetra-acyl sulfoglycolipid, (**B**) diacyl sulfoglycolipid, (**C**) diacyl trehalose, (**D**) triacyl trehalose, (**E**) polyacyl trehalose, (**F**) diacylglycerol, (**G**) triacylglycerol, (**H**) phthiocerol dimycocerosate, (**I**) menaquinone, (**J**) cardiolipin, (**K**) phosphatidyl ethanolamine, (**L**) lyso phosphatidylethanolamine, (**M**) phosphatidyl inositol, (**N**) diacyl phosphatidylinositol dimannoside, (**O**) monoacyl phosphatidylinositol dimannoside, and (**P**) tuberculosinyl adenosine. A representative chemical structure and overlay of

bioRxiv preprint doi: https://doi.org/10.1101/2025.11.06.685907; this version posted November 7, 2025. The copyright holder for this preprint (which was not certified by peer review) is the author/funder, who has granted bioRxiv a license to display the preprint in perpetuity. It is made available under aCC-BY 4.0 International license.

fragments detected for previously unknown (**Q**) lysyldiacylglycerol, identified in these studies from the ‘lipidome of unknowns’.

bioRxiv preprint doi: https://doi.org/10.1101/2025.11.06.685907; this version posted November 7, 2025. The copyright holder for this preprint (which was not certified by peer review) is the author/funder, who has granted bioRxiv a license to display the preprint in perpetuity. It is made available under aCC-BY 4.0 International license.

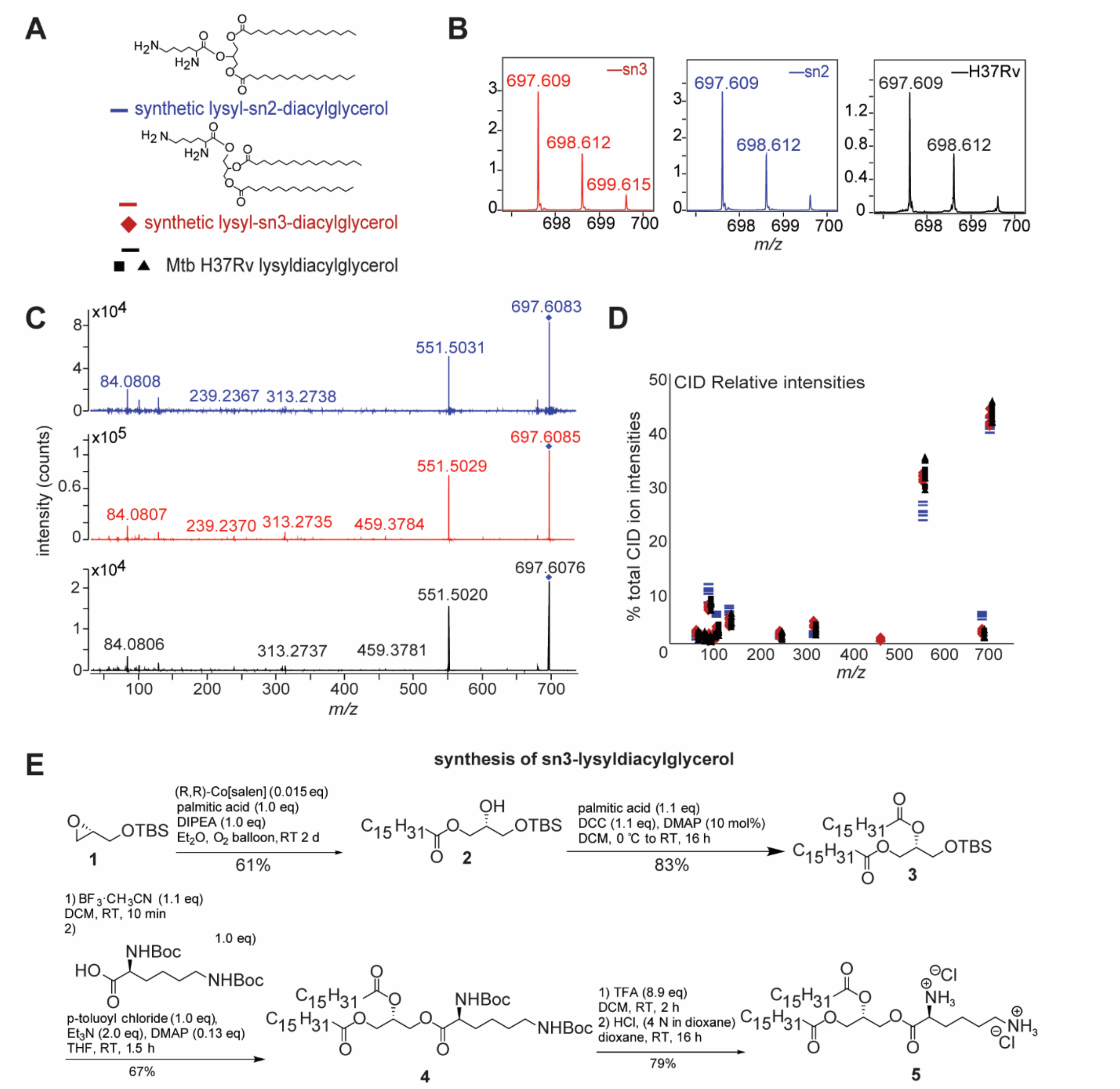

1008

1009 **Fig. S4. Comparison of synthesized and natural lysyldiacylglycerol.** (**A**) Chemical structures of
1010 synthesized lysydiacylglycerol with the lysine head group at the *sn2* (*blue*) or *sn3* (*red*) position serve as a
1011 legend for panels C to F below. (**B**) Mass spectra of synthesized and natural lysyldiacylglycerol show
1012 identical detected masses and isotope distributions and (**C**) identical CID-MS fragments between
1013 synthesized isomers and the natural product (< 10 ppm). However, (**D**) the proportion of intensities of
1014 fragments of the Mtb H37Rv lysyldiacylglycerol (*black*) matches the synthetic *sn3* isomer (*red*) and not the
1015 *sn2* isomer (*blue*) further confirming the natural product structure. (**E**) A simplified chemical synthesis for
1016 *sn3* dipalmityl lysyldiacylglycerol is shown, with representative structures of intermediates as SI Methods.

bioRxiv preprint doi: https://doi.org/10.1101/2025.11.06.685907; this version posted November 7, 2025. The copyright holder for this preprint (which was not certified by peer review) is the author/funder, who has granted bioRxiv a license to display the preprint in perpetuity. It is made available under aCC-BY 4.0 International license.

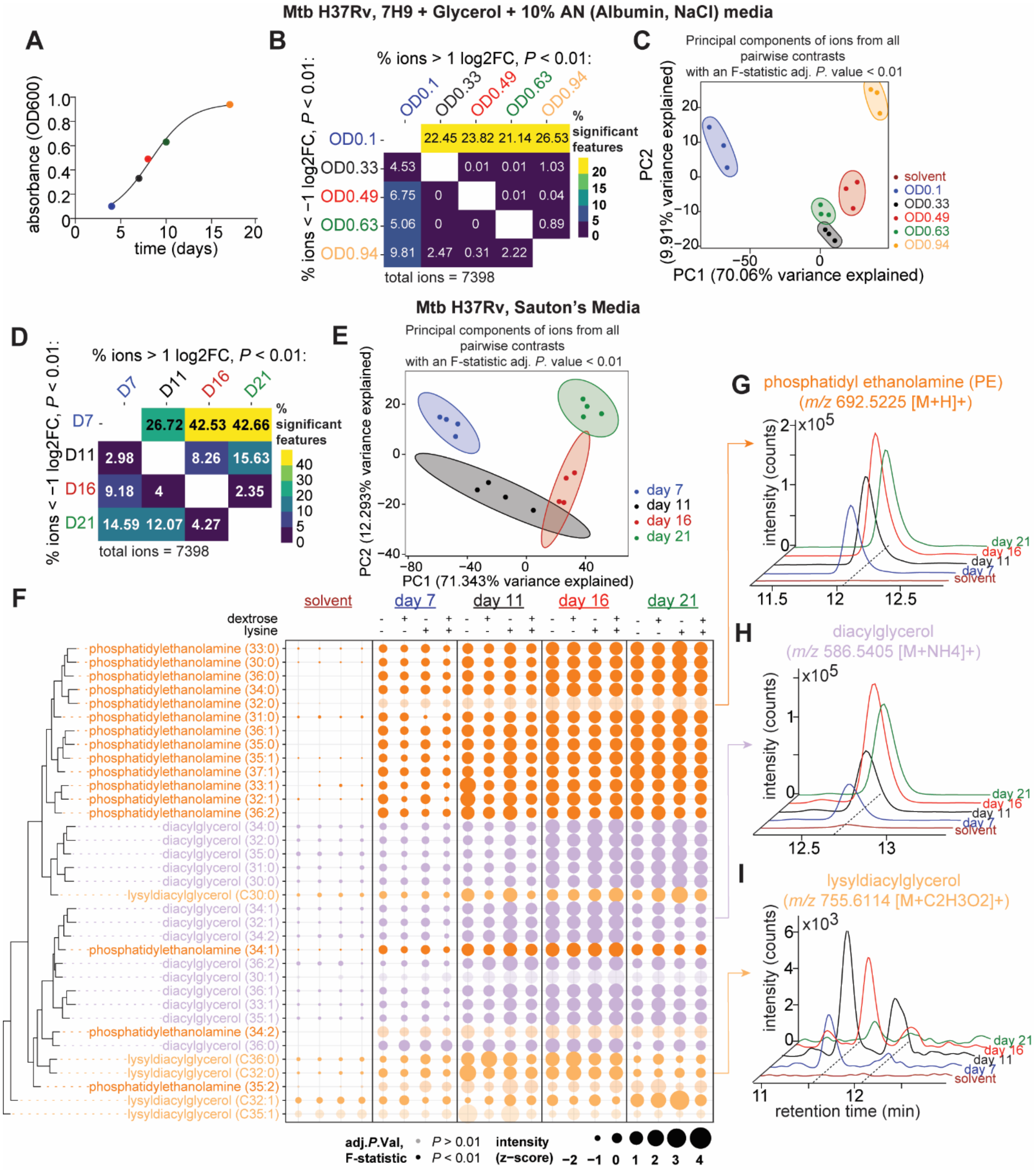

Fig. S5. Identification of lysyldiacylglycerol in 7H9 and Sauton's media. Mtb H37Rv was grown in (**A** to **C**) matched triplicate cultures in 7H9 media with 10% Albumin and NaCl or (**D** to **H**) in detergent-free Sauton's media with glycerol as a sole carbon source in parallel quadruplicate cultures supplemented with either dextrose, lysine or both. (**A**) A matched 0.05% Tween-80 culture in dextrose-free 7H9 was used to determine the optical density at 600 nm at various timepoints as a measure of growth phase. (**B** and **D**) A lipidomic contrast of all pairwise comparisons identified the number of significant lipids with > two-fold change and a Benjamini-Hochberg adjusted *P* value < 0.01 contrasting each time point sampled. These non-solvent contrasts informed the F-statistic used to determine a significance threshold for variability in Figure

bioRxiv preprint doi: https://doi.org/10.1101/2025.11.06.685907; this version posted November 7, 2025. The copyright holder for this preprint (which was not certified by peer review) is the author/funder, who has granted bioRxiv a license to display the preprint in perpetuity. It is made available under aCC-BY 4.0 International license.

2L and Figure S5F respectively. (**C** and **E**) The variance between timepoints was explained using principal components analysis of lipids meeting the significance threshold for variation, *P* value of the F-statistic < 0.01. (**F**) The z-score intensities plotted as bubbles showed the distribution of known acylforms of glycerolipids across growth timepoints in dextrose-free Sauton's media. A significant *P* value of the F-statistic < 0.01, identified variable lipids across growth phase as darkened circles, with representative single ion chromatograms of (**G**) phosphatidyl ethanolamine, (**H**) diacylglycerol, and (**I**) lysyldiacylglycerol.

bioRxiv preprint doi: https://doi.org/10.1101/2025.11.06.685907; this version posted November 7, 2025. The copyright holder for this preprint (which was not certified by peer review) is the author/funder, who has granted bioRxiv a license to display the preprint in perpetuity. It is made available under aCC-BY 4.0 International license.

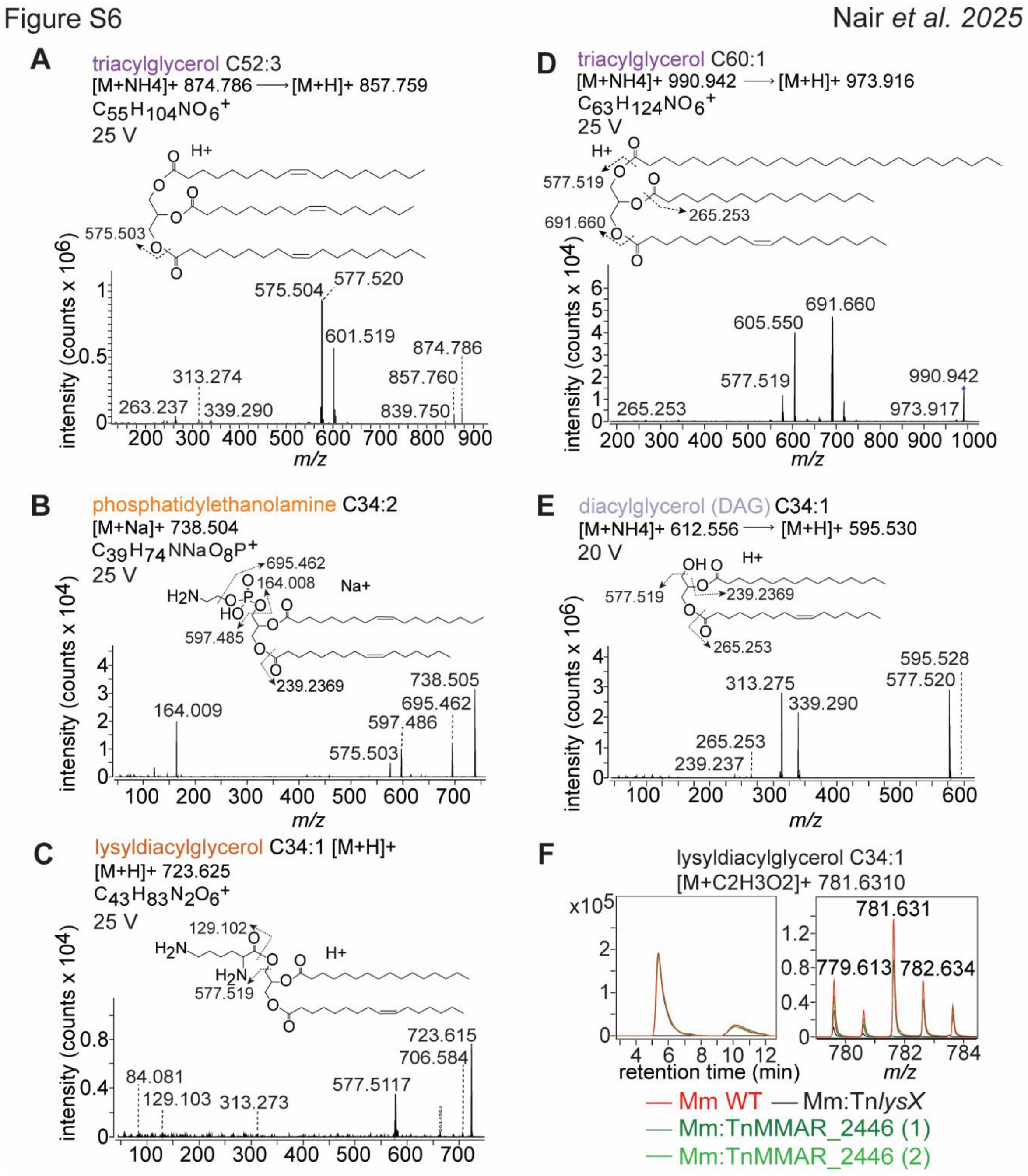

Fig. S6. Collisional MS of *M. marinum* lipids. Collisional MS spectrum and the interpreted fragments overlaid on a representative molecular structure for (**A** and **D**) triacylglycerol, (**B**) phosphatidylethanolamine, (**C**) lysyldiacylglycerol, and (**E**) diacylglycerol in the positive modes with collisional energy indicated for each species identified in the *M. marinum lysX*-dependent lipidome. (**F**) Disruption of the *lysX* downstream gene Rv1639c/MMAR_2246, did not affect levels of lysylDAG represented as chromatograms in the normal phase (*left*) or mass spectrum (*right*), representative of biological quadruplicate cultures. Two independent TnMMAR_2446 mutants were tested.

bioRxiv preprint doi: https://doi.org/10.1101/2025.11.06.685907; this version posted November 7, 2025. The copyright holder for this preprint (which was not certified by peer review) is the author/funder, who has granted bioRxiv a license to display the preprint in perpetuity. It is made available under aCC-BY 4.0 International license.

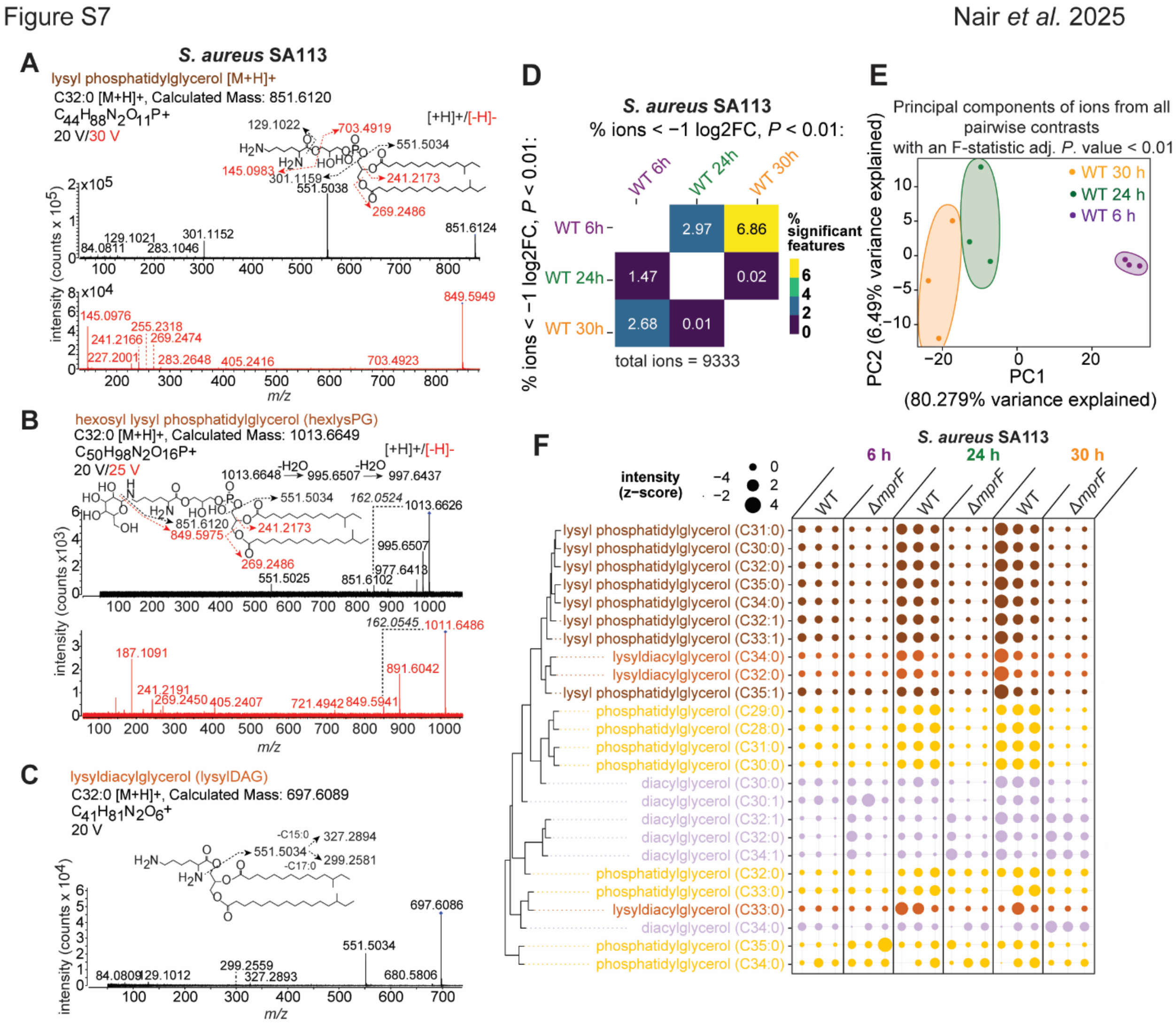

Fig. S7. Collisional MS spectra for *mprF*-dependent lipids. Collisional MS spectrum and the interpreted fragments overlaid on a representative molecular structure in the positive (*black*) and negative modes (*red*) for the C32:0 acylforms of (**A**) lysyl phosphatidylglycerol, (**B**) hexosyl lysyl phosphatidylglycerol, and (**C**) lysyldiacylglycerol in *S. aureus* SA113 lipid extracts. (**D**) A tileplot of all pairwise contrasts between SA 113 WT strains grown at 3 timepoints show the percentage of significant lipids enriched in each phase. (**E**) The variance between timepoints was explained using principal components analysis of lipids meeting the significance threshold for variation, F-statistic adj. *P*. value < 0.01. (**F**) The z-score intensity of lysine and neutral glycerolipids in *S. aureus* SA113 shows *mprF*-dependence of both 8 acylforms of lysylPG and 3 lysylDAG across 3 timepoints in biological triplicate, in contrast to 6 diacylglycerol (DAG).

bioRxiv preprint doi: https://doi.org/10.1101/2025.11.06.685907; this version posted November 7, 2025. The copyright holder for this preprint (which was not certified by peer review) is the author/funder, who has granted bioRxiv a license to display the preprint in perpetuity. It is made available under aCC-BY 4.0 International license.

Data S1. Replication R-Markdown

R markdown (lines 1 - 2145) and 6 supporting csv files, 10 phenotype objects, 12 *xcms* objects, and 1 table, required with formatted datasets S2-S13 to generate all R-based analysis and figures, including the feature credentialling pipeline. A second R markdown includes 4 custom functions to interface with R packages *xcms* and *limms*, and a new function *mzrtMeta* to align mass spectrometry experiments sourced by the replication markdown (available at: https://github.com/jamayfie/mzrtMatch). Users must modify path statements to their own data drive with downloaded data files below. Additionally all R packages, dependencies, and custom functions must be sourced and loaded.

Data S2. MycoMassDB

Mycobacterial database of validated lipids and metabolites, including newly discovered lysine lipoamino acids, formatted for automated matching.

Data S3. MycoLOBSTAH

Mycobacterial database of theoretical lipids in the positive mode propagated from MycoMassDB using LOBSTAHs R-package.

Data S4. Credentialed map of known mycobacterial lipids and lysyldiacylglycerols

Map of Mtb H37Rv qualified features in two groups: known lipids with identities confirmed by collisional MS, and unknown features ranked by quality criteria.

Data S5. *lysX*/*mprF* domain ontology

M. tuberculosis genes with homology to firmicute *mprF*, including their subdomains and Interpro classifications.

Data S6. Lipids knocked down with lysX silencing in Mtb H37Rv

Lipids significantly enriched in the Mtb H37Rv *lysX* CRISPRi knockdown uninduced (-ATc) condition compared to the induced strain (+ATc).

Data S7. Mtb H37Rv growth phase glycerolipids, 7H9

Acylform intensities of diacylglycerol, lysyldiacylglycerol and phosphatidylethanolamine detected across growth phases in Mtb H37Rv grown in 7H9 media.

Data S8. Mtb H37Rv growth phase glycerolipids, Sauton's

bioRxiv preprint doi: https://doi.org/10.1101/2025.11.06.685907; this version posted November 7, 2025. The copyright holder for this preprint (which was not certified by peer review) is the author/funder, who has granted bioRxiv a license to display the preprint in perpetuity. It is made available under aCC-BY 4.0 International license.

Acylform intensities of diacylglycerol, lysyldiacylglycerol and phosphatidylethanolamine detected across growth phases in Mtb H37Rv grown in Sauton's media.

Data S9. Distribution of lysylDAG and other glycerolipids across mycobacteria

Aligned dataset of acylform intensities of diacylglycerol, lysyldiacylglycerol and phosphatidylethanolamine across 15 mycobacterial strains and species.

Data S10. Lipids enriched in the Mm *lysX* mutant

Lipids meeting significance criteria as enriched in the Mm *lysX* mutant from a compound contrast against Mm WT and complement.

Data S11. Lipids enriched in Mm WT and *lysX* complement

Lipids meeting significance criteria as enriched in the Mm WT and *lysX* complement from a compound contrast against the Mm *lysX* mutant.

Data S12. *mprF*-dependent lipids in *S. aureus*

Lipids meeting significance criteria as enriched in *S. aureus* WT compared to the Δ*mprF* mutant

Data S13. *S. aureus* SA113 growth phase glycerolipids

Acylform intensities of diacylglycerol, lysyldiacylglycerol and lysyl phosphatidylglycerol detected across growth phases in *S. aureus* SA 113 and the Δ*mprF* mutant
